# Host genetics and gut microbiota synergistically regulate feed utilization in egg-type chickens

**DOI:** 10.1186/s40104-024-01076-7

**Published:** 2024-09-09

**Authors:** Wenxin Zhang, Fangren Lan, Qianqian Zhou, Shuang Gu, Xiaochang Li, Chaoliang Wen, Ning Yang, Congjiao Sun

**Affiliations:** State Key Laboratory of Animal Biotech Breeding and Frontier Science Center for Molecular Design Breeding, China Agricultural University, Beijing, 100193 China

**Keywords:** Feed efficiency, Genetic variations, Gut microbiota, Laying hens, Multi-omics

## Abstract

**Background:**

Feed efficiency is a crucial economic trait in poultry industry. Both host genetics and gut microbiota influence feed efficiency. However, the associations between gut microbiota and host genetics, as well as their combined contributions to feed efficiency in laying hens during the late laying period, remain largely unclear.

**Methods:**

In total, 686 laying hens were used for whole-genome resequencing and liver transcriptome sequencing. 16S rRNA gene sequencing was conducted on gut chyme (duodenum, jejunum, ileum, and cecum) and fecal samples from 705 individuals. Bioinformatic analysis was performed by integrating the genome, transcriptome, and microbiome to screen for key genetic variations, genes, and gut microbiota associated with feed efficiency.

**Results:**

The heritability of feed conversion ratio (FCR) and residual feed intake (RFI) was determined to be 0.28 and 0.48, respectively. The ileal and fecal microbiota accounted for 15% and 10% of the FCR variance, while the jejunal, cecal, and fecal microbiota accounted for 20%, 11%, and 10% of the RFI variance. Through SMR analysis based on summary data from liver eQTL mapping and GWAS, we further identified four protein-coding genes, *SUCLA2*, *TNFSF13B*, *SERTM1*, and *MARVELD3*, that influence feed efficiency in laying hens. The *SUCLA2* and *TNFSF13B* genes were significantly associated with SNP 1:25664581 and SNP rs312433097, respectively. *SERTM1* showed significant associations with rs730958360 and 1:33542680 and is a potential causal gene associated with the abundance of Corynebacteriaceae in feces. *MARVELD3* was significantly associated with the 1:135348198 and was significantly correlated with the abundance of *Enterococcus* in ileum. Specifically, a lower abundance of *Enterococcus* in ileum and a higher abundance of Corynebacteriaceae in feces were associated with better feed efficiency.

**Conclusions:**

This study confirms that both host genetics and gut microbiota can drive variations in feed efficiency. A small portion of the gut microbiota often interacts with host genes, collectively enhancing feed efficiency. Therefore, targeting both the gut microbiota and host genetic variation by supporting more efficient taxa and selective breeding could improve feed efficiency in laying hens during the late laying period.

**Supplementary Information:**

The online version contains supplementary material available at 10.1186/s40104-024-01076-7.

## Introduction

Feed efficiency is a crucial trait for poultry production, as it plays a significant role in reducing overall feed consumption, consequently decreasing farm costs and minimizing environmental impacts [1, 2]. Feed conversion ratio (FCR) and residual feed intake (RFI) serve as key indicators for evaluating feed efficiency in livestock. Specifically, FCR of egg-type chickens is defined as the ratio of feed intake (FI) to egg mass during a specified measuring period [3]. RFI, initially proposed by Koch et al. [4] for cattle, is defined as the difference between the actual feed intake of the livestock and the expected feed intake required for maintenance and production. Host genetics plays a crucial role in regulating feed utilization, as numerous studies have reported moderate to high heritability of FCR and RFI in chickens [5–9]. In fact, the breeding for RFI in laying hens has been discussed for over 40 years [8]. Additionally, the chicken gastrointestinal tract (GIT) hosts a diverse microbial community that is essential for nutrient digestion, absorption, and carbohydrate metabolism, especially when processing indigestible polysaccharides [10–12]. Increasing evidence has confirmed a close association between gut microbiota and feed efficiency in chickens. For instance, Yan et al. [13] and Wen et al. [14] showed that the cecal microbiota plays a significant role in the feed efficiency of chickens, and higher abundance of cecal *Lactobacillus* was associated with better feed efficiency. Siegerstetter et al. [15] reported that low-abundance taxa in the ileum, cecum, and feces may have certain effects on the feed efficiency of chickens, with the association between bacteria in the chyme of the ileum and cecum and feed efficiency possibly serving as useful targets for feed strategies. Studies on both humans and animals have suggested that host genetic factors play a role in shaping the gut microbial community. For example, monozygotic twins exhibit more similar microbial structures compared to dizygotic twins and unrelated individuals, indicating the influence of host genetics on the human gut microbiome composition within populations [16, 17]. Furthermore, Wen et al. [18] estimated the SNP heritability of microbiota in various gut segments and feces of broilers, revealing that a small percentage of bacterial genera in each segment and feces exhibited significant heritability, mainly consisting of phyla Firmicutes and Proteobacteria. This suggests that these microbial species or groups have some level of genetic transmission among host individuals. However, the specific contributions of host genetics to shaping the gut microbiota remain unclear, with various research findings showing some contradictions [18, 19]. This indicates a complex interaction between host genetics and the gut microbiome; thus, elucidating the interplay between host genetics and microbiota may provide valuable insights into complex traits.

In recent years, various multi-omics approaches, primarily utilizing high-throughput sequencing technologies, have been widely employed to investigate complex traits in animals [20–22]. Multi-omics approaches provide opportunities to comprehensively understand the fundamental flow of information governing complex traits, in contrast to the single omics type-focused studies [23, 24]. Among these, genome-wide association studies (GWAS) have played a significant role in exploring genomic variations related to complex traits in farm animals [25], and notable discoveries have been made in terms of feed efficiency in chickens [5, 26]. However, GWAS alone is not able to directly establish causal relationships. To address this limitation, researchers have started using the Mendelian randomization (MR) framework, which combines summary statistics from various GWAS to infer causal relationships [27–29]. This method has been successfully applied to identify genes causally linked with complex traits [30–33]. Expression quantitative trait loci (eQTLs), which are genetic variations that influence gene expression levels, offer an alternative research perspective. By applying the summary data-based Mendelian randomization (SMR) method [32], which integrates summary-level data from independent GWAS with data from eQTL studies, we can effectively identify genes whose expression levels are causally linked to feed efficiency in laying hens.

With improvements in the persistence and stability of egg production, the extension of the laying period in hens has become a focal point in both production practices and scientific research [6, 34]. However, as hens progress through their laying cycle, feed efficiency tends to decrease [3, 35], presenting a challenge to the economic viability of poultry farming. Despite its significance, the regulatory mechanism of feed efficiency in laying hens during the late laying period remains unclear, with limited research on the impact of genetic background and gut microbiota [5, 6, 36]. This lack of understanding hinders our ability to pinpoint and enhance traits that could improve feed efficiency in breeding programs. To bridge this gap in knowledge, our study aimed to systematically explore the combined regulatory mechanisms of host genetics and gut microbiota on feed efficiency in laying hens, utilizing a multi-omics approach. Through this research, we sought to gain a more comprehensive understanding of this intricate trait and establish a foundation for more precise and efficient breeding strategies.

## Materials and methods

### Ethics statement

All experiments involving animals were conducted according to the ethical policies and procedures approved by the Institutional Animal Care and Use Committee of China Agricultural University, China (Issue No.32303202-1-1).

### Animals, phenotypic data and sample collection

A total of 725 female purebred Rhode Island Red chickens originating from 87 paternal families were used in the current study. These chickens were provided by Beijing Huadu Yukou Poultry Breeding Co., Ltd. (China). All chickens came from 2 batches with a 10-d interval and were reared in individual cages with free access to feed and water. The illumination schedule followed a photoperiod of 16 h light and 8 h darkness on a daily basis (16 L:8 D). Feed intake and egg production were individually recorded from 69 to 72 weeks of age. The body weight of each chicken at 69 and 72 weeks of age were measured using an electronic scale. Each hen was provided with mash feed on individual metal feeders. The daily feed intake (DFI), daily egg mass (DEM), and daily body weight gain (DBWG) per hen during the trial period were calculated. Metabolic body weight (MBW), feed conversion ratio (FCR), and residual feed intake (RFI) were calculated as described by Yan et al. [37]. The calculation formulas for FCR and RFI are as follows:

$$\mathrm{FCR}\:=\:\mathrm{DFI}/\mathrm{DEM}$$

$$\mathrm{RFI}\:=\:\mathrm{DFI}\:-\:({\mathrm b}_0\:+\:{\mathrm b}_1\:\times\:\mathrm{MBW}\:+\:{\mathrm b}_2\:\times\:\mathrm{DEM}\:+\:{\mathrm b}_3\:\times\:\mathrm{DBWG})$$

Where b_0_ is the intercept, b_1_, b_2_, and b_3_ are the partial regression coefficients. Normality for all the traits was checked using the Shapiro–Wilk test in the R program (ver 4.3.1). The descriptive statistics of these phenotypes are summarized in Additional file 1: Table S1. The correlation coefficient between RFI and DFI was 0.76, and the correlation of RFI with DEM, DBWG and MBW was negligible. In addition, FCR and DEM showed a high phenotypic negative correlation with a correlation coefficient of −0.67 (Additional file 2: Fig. S1).

Whole blood was collected from each bird via the wing vein using a syringe, and fecal samples were manually collected from the cloaca with sterile cotton swabs. The hens were euthanized by cervical dislocation. The duodenal, jejunal, ileal, and cecal contents (including chyme and mucosa) and liver tissue were collected immediately. All samples were snap-frozen in liquid nitrogen and stored at −80 °C for subsequent studies.

### Whole-genome resequencing and data processing

The host genomic DNA was isolated from liver samples using the Tiangen DNA Extraction Kit (Tiangen Biotech, Beijing, China). A total of 686 host DNA samples were sequenced using an Illumina HiSeq 2500 Sequencer (Illumina, Inc., San Diego, CA, USA) to generate 150 bp paired-end reads. To avoid artificial bias, low-quality reads were removed using FastQC (version 11.7). The clean reads were then mapped to the chicken reference genome (GRCg6a) using BWA (ver 0.7.15) [38] and sorted with Samtools (ver 1.3.1) [39]. Duplicates were removed using Picard tools (http://broadinstitute.github.io/picard/), and SNP calling and genotyping were performed using the HaplotypeCaller module in the Genome Analysis Toolkit (GATK, ver 4.2.0.0) [40]. The SNPs were then filtered using the GATK VariantFiltration module as follows: QD < 2.0, ReadPosRankSum <  −8.0, FS > 60.0, QUAL < 30.0, DP < 4.0, MQ < 40.0, MappingQualityRankSum <  −12.5 and INDEL: QD < 2.0, ReadPosRankSum <  −20.0, FS > 200.0, QUAL < 30.0, DP < 4.0. Finally, the annotated SNP data were filtered using PLINK (ver 1.90) [41] with the following parameters: sample call rate > 90%, SNP call rate > 90%, and minor allele frequency > 1%. The remaining SNPs and individuals were used for imputation in BEAGLE (ver 5.1) [42]. After reanalyzing the same criteria using PLINK, a total of 5,904,820 SNPs were retained for subsequent analysis.

### RNA-seq and data analysis

A total of 686 liver samples were used for RNA-seq. Total RNA was extracted using the Eastep^®^ Super Total RNA Extraction Kit (Promega, Shanghai, China, LS1040) according to the manufacturer’s instructions. After determining RNA concentration and purity and assessing RNA integrity, transcriptome sequencing libraries were constructed according to the standard Illumina RNA-seq protocol and sequenced on the Illumina Novaseq platform, generating 150 bp paired-end reads. Fastp (ver 0.20.1) [43] was used to remove reads containing adapter contamination, low-quality bases, and undetermined bases. Clean reads were aligned to the chicken reference genome (GRCg6a) using HISAT2 (ver 2.0.5) [44] with default parameters. Subsequently, featureCounts (ver 1.6.3) [45] was employed to count the reads for each gene. The obtained count matrix was used for subsequent analysis of differentially expressed genes (DEGs).

### 16S rRNA gene sequencing and analysis

Microbial DNA from the gut chyme (duodenum, jejunum, ileum, and cecum) and fecal samples of 705 individuals were extracted using the QIAamp DNA Stool Mini Kit (QIAGEN, Hilden, Germany, D4015-01) following the manufacturer’s instructions. PCR amplification of the V4 region (515F-806R) of the 16S rRNA gene was performed. The PCR reactions were performed in a 30-μL system containing 15 µL of Phusion High-Fidelity PCR Master Mix (New England Biolabs, Ipswich, MA, USA), 0.2 µmol/L forward primer, 0.2 µmol/L reverse primer, and 10 ng template DNA. The optimum PCR program was as follows: 98 °C for 1 min, 30 cycles of 98 °C for 10 s, 50 °C for 30 s, 72 °C for 30 s, and a final extension at 72 °C for 5 min. Finally, 3,519 microbial DNA samples were sequenced. The V4 hypervariable region of the 16S rRNA gene was amplified using the Ion Plus Fragment Library Kit 48 rxns (Thermo Scientific) and sequenced on the Ion S5TM XL platform (400 bp single-end reads). The obtained sequences were subjected to data QC and bioinformatic analysis using Quantitative Insights Into Microbial Ecology (QIIME2, ver 2019.10) [46]. After trimming the barcode and primer sequences, the preliminary quality screening was performed for the raw high-throughput sequencing data using the QIIME2 plugin DADA2 [47]. The chimeric sequences were filtered and the remaining sequences were trimmed to a final length of 252 bp. The remaining high-quality sequences were aligned and clustered into amplicon sequence variants (ASVs) with 100% sequence identity [48]. ASVs appearing in less than 1% (7) of samples and with an average relative abundance below 10^−6^ were removed. Annotation for each ASV was performed using the SILVA 16S rRNA gene sequence reference database (Release 132) [49].

### Statistical analysis

To detect specific genes that significantly influence feed efficiency, in R version 4.3.1 [50], the psych package in R was used to calculate the Spearman correlation coefficients between Transcripts Per Million (TPM) values, FCR, and RFI. Simultaneously, Analysis of variance (ANOVA) was employed to analyze the differences in liver gene expression (TPM) between the top and bottom 20% of chickens based on FCR and RFI. As previously reported [18, 51], the microbial taxa with low detection rates were not statistically meaningful. Therefore, only taxa with detection rates ≥ 30% in each gut segment or fecal sample were retained. For the remaining genera, Spearman rank correlation coefficients between the genus-level relative abundance of the microbial community and FCR and RFI were computed in the jejunum, ileum, cecum and feces. To identify the microbes significantly associated with feed efficiency, individuals in the top and bottom 20% of the phenotype were selected. Wilcoxon rank-sum tests were then conducted on the relative abundance of various taxa, retaining only those microbial taxa with a detection rate ≥ 30% in each intestinal segment or fecal sample. Linear discriminant analysis Effect Size (LEfSe) analysis was further conducted on the relative abundances of all detected microbes to determine significantly different taxa.

For SNPs or genomic regions significantly associated with feed efficiency, we extracted the genotypes at each locus and analyzed differences in liver gene expression (TPM), FCR, and RFI among individuals with different genotypes using the Wilcoxon rank-sum and Kruskal–Wallis tests. *P*-values were corrected using the false discovery rate (FDR) method. For SNPs or genomic regions significantly associated with the abundance of key differential microbial genera, we extracted the genotypes at each locus and analyzed differences in liver gene expression (TPM) and microbial genus abundance among individuals with different genotypes using the Wilcoxon rank-sum and Kruskal–Wallis tests. The *P*-values were corrected using the FDR method. Additionally, to detect specific microbes significantly influencing feed efficiency, the Wilcoxon rank-sum test was employed to assess differences in FCR and RFI between the top and bottom 20% of chickens based on the relative abundance of specific microbial genera.

#### Evaluating effects of host genetics and the gut microbiota on feed efficiency

To estimate the contribution of host genetics to feed efficiency, we employed the following model in GCTA software (ver 1.93.2) [52] for restricted maximum likelihood analysis to estimate the SNP-based heritability for feed efficiency:

$$\boldsymbol{Y}\:=\:\boldsymbol{Kc}\:+\:\boldsymbol{g}\:+\:\boldsymbol{e}$$

Here, ***Y*** is a vector of phenotypes; ***K*** is a design matrix of covariates, including batch effect and the first ten principal components of host genetics; ***c*** is a vector of fixed covariate effects; ***g*** is a vector of polygenic effects following the normal distribution *N *(0, **G**σ^2^_*g*_), where ***G*** is the genetic relatedness matrix (GRM) calculated based on genome-wide marker information, σ^2^_*g*_ is the genetic variance, and ***e*** is a vector of residual errors. Similarly, the GRM was constructed using GCTA software (version 1.93.2) [52] based on the following model:

$$g_{ij}\:=\frac1N\sum\nolimits_{V=1}^N\frac{\left(x_{iv}-2\overline{p_v}\right)\left(x_{jv}-2\overline{p_v}\right)}{2\overline{p_v}\left(1-\overline{p_v}\right)}$$

where *g*_*ij*_ represents the genetic relationship between individuals *i* and *j*, *x*_*iv*_ and *x*_*jv*_ represent the number of reference alleles for individuals *i* and *j*, respectively, *p*_*v*_​ represents the reference allele frequency, and *N* is the number of SNP. Additionally, to ascertain the effect of host genetics on a specific taxon, the above model was also employed to estimate the heritability of the gut microbiota. As described in previous studies [14, 18, 51], microbial genera with a detection rate of less than 30% in the samples were excluded. Only microbial genera with a detection rate of ≥ 30% were retained for heritability estimation. Microbial genera with detection rates between 30% and 60% were treated as binary traits (coded 0 or 1). The relative abundances of microbial genera with a detection rate greater than 60% were considered quantitative traits. Among microbial genera with a detection rate greater than 60%, those with a relative abundance of 0 were converted to NA. And the Shapiro–Wilk normality test was conducted. For microbial genera that did not follow a normal distribution, the GenABEL package in R was used to perform a normal transformation of their relative abundances. Vector ***y*** in the aforementioned model parameters was then replaced with the vector of corrected phenotypes (abundance or presence/absence of microbial genera), and the SNP heritability of each microbial genus was estimated individually.

To assess the proportion of variation in feed efficiency attributed to the microbiota from different sampling sites, as described in our previous research [18], we constructed a microbial relationship matrix (MRM) using GCTA software and fitted a model to estimate the phenotypic variance explained by the microbial community. In this analysis, we corrected for batch effects and the first five host genetic principal components. The phenotypic variance explained by gut microbial variance is called microbiability (*m*^2^) in animals [53–55], calculated as *m*^2^ = σ^2^m/σ^2^p, where σ^2^m is the microbial variance.

#### Genome-wide association study

The phenotypes of feed conversion ratio (FCR) and residual feed intake (RFI) were subjected to a Shapiro–Wilk normality test, and the GenABEL package in R [56] was employed for normality transformation. Subsequently, the following univariate linear mixed model (LMM), implemented in GEMMA (ver 0.98.4) [57], was employed for genome-wide association study (GWAS) to detect significant host genetic markers influencing feed efficiency: 

$$\boldsymbol{y}\:=\:\boldsymbol{W\alpha}\:+\:\boldsymbol{X}\beta\:+\:\boldsymbol{u}\:+\:\boldsymbol{\varepsilon}$$

where ***y*** is a vector of corrected phenotypes; ***W*** is a matrix of covariates controlling for population structure, including the top five host genetic principal components and batch effects; ***α*** is a vector of effects for the covariates (including the intercept); ***X*** is a vector of allele counts (0, 1, 2); and *β* is the SNP effect; ***u*** is a vector of random polygenic effects with a covariance structure; ***ε*** is a vector of residual errors. The likelihood ratio test was conducted to calculate *P*-values for the SNP effects. Due to the conservative nature of the traditional Bonferroni correction, the effective number of independent tests was computed using simpleM package in R [58], resulting in 150,802 independent tests. Consequently, the genome-wide significance threshold was set at 3.32 × 10^−7^ (0.05/150,802), and the suggestive significance threshold was set at 6.63 × 10^−6^ (1/150,802). Additionally, we further performed mGWAS [14, 59] analysis using the aforementioned linear mixed model in GEMMA to identify crucial host genetic markers influencing differential microbial genera. The first five host genetic principal components were included as covariates. Likelihood ratio tests were used for significance testing. The significance threshold and suggestive significance threshold were set as described above.

To elucidate the impact of host genetics on gene expression in the liver, fastGWA [60] analysis was conducted using Genome-Wide Complex Trait Analysis (GCTA) software (ver 1.93.2) [52]. A total of 668 experimental subjects with transcriptome data were used for the subsequent genomic and gene expression association analyses. Read counts were normalized using TPM. Genes with a detection rate of ≥ 20% were selected based on the thresholds of TPM ≥ 0.1 and reads ≥ 6 (non-normalized). Subsequently, the TMM method was applied to normalize the read counts between samples. Finally, 12,191 genes were retained. For fastGWA, a full-dense genetic relationship matrix (GRM) was first generated, and a sparse GRM was constructed with a cutoff value of 0.05. Following this, the sparse GRM was used in fastGWA based on the aforementioned LMM, with gene expression levels as the dependent variable and SNP genotypic values as the independent variable. As mentioned above, the significance and suggestive significance thresholds were set to 3.32 × 10^−7^ and 6.63 × 10^−6^, respectively.

#### eQTL mapping and summary data-based Mendelian randomization analysis

The eQTL mapping framework includes 2 main phases: data preprocessing and eQTL mapping. Genes were filtered based on expression levels, considering TPM > 0.1 or read counts > 6 in ≥ 20% of the samples. TMM normalization was applied to TPM values within samples. We employed TensorQTL to perform *cis*- and *trans*-eQTL mapping [61]. We considered all genetic variants and used the top 5 genetic principal components, batch effects, and the top 3 PEER factors as covariates. The number of calculated PEER factors was determined based on previously reported correspondences with sample sizes (15 for *n* < 150, 30 for 150 ≤ *n* < 250, 45 for 250 ≤ *n* < 350, and 60 for *n* ≥ 350) [62]. Subsequently, permutation was performed for *cis*-eQTL mapping to generate empirical *P*-values for summary statistics of phenotype levels, and *trans*-eQTL mapping was conducted to compute nominal associations between all phenotypes and genotypes. For *cis*-eQTL analysis, SNPs were included if their positions were within 1 Mb of the transcription start site (TSS) of the gene, whereas for *trans*-eQTL analysis, SNPs were filtered using stringent quality control criteria, requiring a minor allele frequency (MAF) > 5% and being outside the TSS ± 5 Mb, resulting in a final set of 5,315,471 common genetic variants. The FDR reported using the Benjamini–Hochberg procedure was employed to measure the significance of associations. The *P*-value threshold for *cis*-eQTL was set at 8.05 × 10^−6^, and for *trans*-eQTL, it was set at 3.32 × 10^−7^.

Summary data-based Mendelian randomization (SMR) [32] is a method that integrates summary statistics from GWAS and eQTL studies within the Mendelian randomization (MR) framework, prioritizing genes for which there may be a causal relationship between expression levels and an outcome trait. We employed the SMR method for the analysis of the top eQTLs. The SMR procedure involves 2 main steps: i) identifying variants independently associated with the exposure factor, and ii) calculating causal estimates. Initially, a BESD file was generated, updating the coordinates of SNPs and genes as well as the frequency of the effect allele. Subsequently, SNPs significantly and suggestively significantly associated with the trait in the GWAS summary statistics were selected as input files to identify connections with significant *cis*-eQTLs and *trans*-eQTLs. Finally, variants were selected based on the association threshold with an FDR-corrected *P*-value < 0.05. We also compiled summary statistics of significant or suggestive significant SNPs from microbial genome-wide association studies (mGWAS) as input files. These were integrated with summary statistics from eQTL studies for the SMR test, prioritizing genes for which a causal relationship between expression levels and microbial genus abundance is likely. Variants were selected based on an association threshold with FDR-corrected *P*-values < 0.05.

## Results

### Characterization of host phenotype and output of sequencing data

The descriptive statistics of the host phenotypes are summarized in Additional file 1: Table S1. Except for MBW, none of the phenotypes observed in our study followed a normal distribution (Shapiro–Wilk test, *P* > 0.05). A strong negative phenotypic correlation was found between FCR and DEM (*r* =  −0.67, *P* < 0.001). Additionally, a significant phenotypic correlation was observed between RFI and DFI (*r* = 0.76, *P* < 0.001). However, the correlations between RFI and MBW, DEM, and DBWG were minimal, and the correlation between RFI and FCR was 0.27 (*P* < 0.001, Additional file 2: Fig. S1).

To obtain the host genomic variants, we generated a total of 7.73 Tb of clean bases from 686 chickens, achieving an average sequencing depth of 8.67-fold with a genome coverage of 95.06%. After stringent quality control, we identified a total of 5,904,820 SNPs (6.17 SNPs/kb) (Additional file 1: Table S2). To analyze the host gene expression, we conducted transcriptome sequencing. A total of 14,969,609,834 raw reads were generated from 668 liver samples. After quality control, 14,174,996,287 high-quality reads were obtained. The clean data of 668 liver samples varied from 5.24 to 10.51 G per individual, and the expression of 12,191 genes was quantified, with 10,171 genes successfully annotated. 16S rRNA gene sequencing yielded a total of 174.2 million quality-filtered sequences from 3,519 samples, with an average of 49,497 reads per sample (Additional file 1: Table S3). Subsequent analysis based on 100% sequence identity revealed 6,087 ASVs in the duodenum, 5,987 in the jejunum, 3,751 in the ileum, 3,215 in the cecum, and 7,428 in the feces, which were classified into 3,930 species, 2,329 genera, 1,003 families, 467 orders, 161 classes, and 52 phyla (Additional file 3: Fig. S2).

### Proportion of variation in feed efficiency explained by host genetics and gut microbiota

**Fig. 1:**
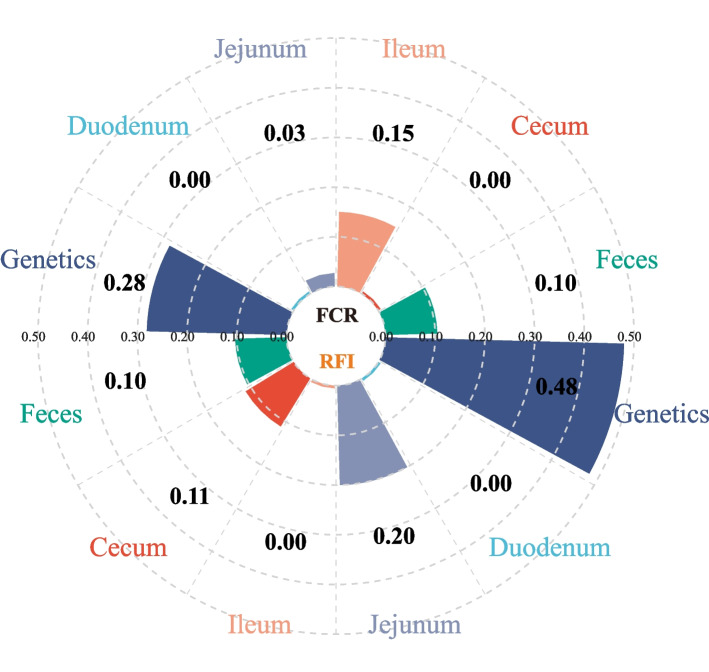
Heritability and microbiability of feed conversion ratio (FCR) and residual feed intake (RFI)

Heritability (*h*^2^) and microbiability (*m*^2^) were calculated to represent the contribution of host genetics and gut microbiota to the phenotypic variance of feed efficiency. The SNP based *h*^2^ of FCR and RFI was found to be 0.28 and 0.48 (Fig. 1) respectively, indicating a medium and high levels of host genetic control for feed efficiency traits. The ASV-based *m*^2^ was then calculated for each gut segment, during which the first five host genetic principal components were included in the estimation model as covariables to correct the effects of host genetics. The results showed that the estimated *m*^2^ using ileal and fecal microbial ASV data are 0.15 and 0.1 respectively (*P* < 0.1), while the *m*^2^ of the FCR estimated by the microbiota in the duodenum, jejunum, and cecum were 0, 0.03, and 0, respectively (Fig. 1). Similarly, the *m*^2^ of the RFI for jejunum, cecum, and feces were 0.20, 0.11, and 0.10, respectively, while the *m*^2^ of the RFI for duodenum and ileum was 0 (Fig. 1). These results indicated that the ileal and fecal microbiota were more closely associated with FCR, whereas the microbiota in the jejunum, cecum and feces were more closely linked to RFI.

### Identification of host genomic variants and liver eQTLs associated with feed efficiency

**Fig. 2:**
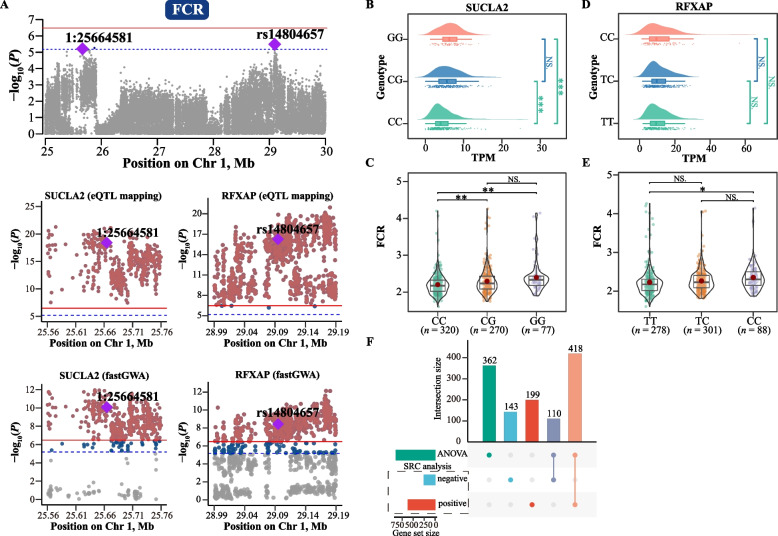
FCR-related eVariants and candidate causal genes. **A** Genome-wide association analysis of feed conversion ratio (FCR). The horizontal red solid line and blue dashed line indicate genome-wide significance (*P* = 3.32 × 10^−7^) and suggestive significance (*P* = 6.63 × 10^−6^) thresholds, respectively. Gray, dark blue and reddish-brown dots indicate non-significant, suggestively significant and significant SNPs, respectively. Colocalization analysis of *trans*-eQTLs for *SUCLA2* gene and *RFXAP* gene in chicken liver, along with GWAS loci for FCR on chromosome 1, identified 2 colocalized SNPs, which correspond to significant *trans*-eQTLs for *SUCLA2* and *RFXAP*, as well as suggestively significant GWAS signals for FCR. **B** and** D** The expression levels of *SUCLA2* and *RFXAP* genes in the liver corresponding to the three genotypes of these two eVariant (1:25664581 and rs14804657) were compared. ***, **, *, and NS represent adjusted *P* values < 0.001, < 0.01, < 0.05, and > 0.05, respectively. **C** and** E** Comparison of FCR among the three genotypes of these two eVariant (1:25664581 and rs14804657). Each point represents an individual chicken. The data and central red dots indicate the number and mean values of the corresponding genotypes, respectively. ***, **, *, and NS represent adjusted *P* values < 0.001, < 0.01, < 0.05, and > 0.05, respectively. **F** Candidate genes associated with FCR were identified using Spearman’s rank-based correlation (SRC) analysis and analysis of variance (ANOVA)

**Fig. 3:**
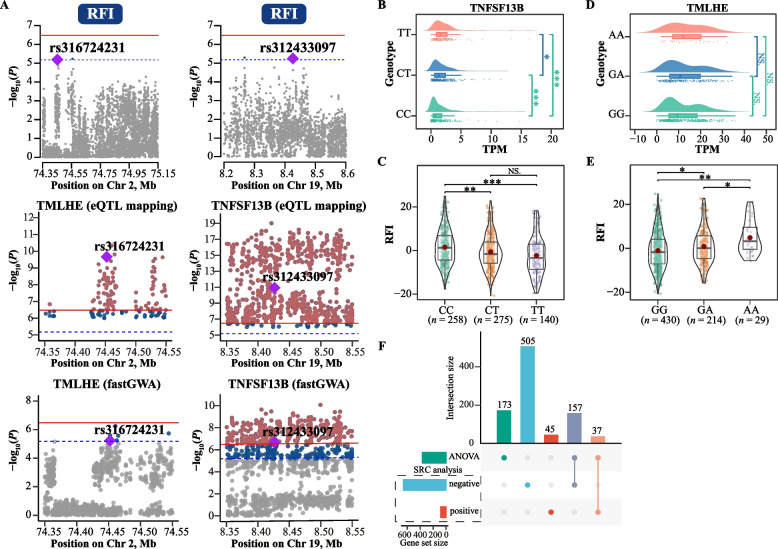
RFI-related eVariants and candidate causal genes. **A** Genome-wide association analysis for residual feed intake (RFI). The horizontal red solid line and blue dashed line indicate genome-wide significance (*P* = 3.32 × 10^−7^) and suggestive significance (*P* = 6.63 × 10^−6^) thresholds. Gray, dark blue and reddish-brown dots indicate non-significant, suggestively significant and significant SNPs, respectively. Colocalization analysis was performed for *trans*-eQTLs of the *TNFSF13B* gene and *TMLHE* gene in the chicken liver, along with GWAS loci for RFI on chromosomes 19 and 2. Two colocalized SNPs were identified, representing significant *trans*-eQTLs for *TNFSF13B* and *TMLHE*, as well as suggestively significant GWAS signals for RFI. **B** and** D** The expression levels of *TNFSF13B* and *TMLHE* genes in the liver corresponding to the 3 genotypes of these 2 variants (rs312433097 and rs316724231) were compared. ***, **, *, and NS represent adjusted *P* values < 0.001, < 0.01, < 0.05, and > 0.05, respectively. **C** and** E** Comparison of RFI among the 3 genotypes of these 2 eVariant (rs312433097 and rs316724231). Each point represents an individual chicken. The data and central red dot respectively indicate the number and mean value of the corresponding genotypes. ***, **, *, and NS represent adjusted *P* values < 0.001, < 0.01, < 0.05, and > 0.05, respectively. **F** Candidate genes associated with RFI were identified using Spearman's rank-based correlation (SRC) analysis and analysis of variance (ANOVA)

Genome-wide association analysis (GWAS) was conducted to further elucidate the regulation of feed efficiency by host genetics. We identified 21 and 8 suggestive significant SNPs associated with FCR and RFI respectively, mainly located on chromosomes 1, 2, 5, 7, and 19 (Fig. 2A and 3A, Additional file 1: Table S4 for FCR and Table S5 for RFI). To determine which genes are regulated by these genetic variants, we used the liver transcriptome data of 686 individuals to conduct eQTL mapping analysis. Using the TensorQTL pipeline, we identified 217 *cis*-regulated genes and 7,431 *trans*-regulated genes. Subsequently, the SMR method was employed to identify the key liver genes for feed efficiency with summary statistics from GWAS and eQTL study. SMR tests revealed that the *RFXAP*, *SUCLA2*, *SERTM1,* and *CAB39L* genes were significantly correlated with genomic variations associated with FCR on GGA1: rs14804657 (1:29094903), 1:25664581 and rs730958360 (1:29111712) (*P*_adj_ < 0.05, FDR correction for SMR, Additional file 1: Table S6). Additionally, *TNFSF13B* and *TMLHE* were significantly associated with genomic variations related to RFI on GGA19: rs312433097 (19:8426112) and GGA2: rs316724231 (2:74452183) (*P*_adj_ < 0.05, FDR correction for SMR, Additional file 1: Table S7). To validate the above associations between genetic variations and gene expression, extensive fastGWA runs were conducted on all liver genes to apply the colocalization strategy. Ultimately, we identified that the expression of 4,171 genes (eGenes) was significantly regulated by at least one genomic variant (*P*-value of the top SNPs < 3.32 × 10^−7^). Among these genes, *SUCLA2* and *RFXAP* on GGA1 were once again identified to be significantly associated with the variants of 1:25664581 and rs14804657 on GGA1, respectively (Fig. 2A). As mentioned earlier, both of these two SNPs (1:25664581 and rs14804657) were also closely associated with FCR. Similarly, *TNFSF13B* was found to be significantly associated with the SNP rs312433097, which also regulates RFI (Fig. 3A).

Furthermore, the expression of the *SUCLA2* gene and FCR exhibited significant differences between individuals corresponding to the CC and G_ genotypes associated with the variation of 1:25664581 (Fig. 2B and C). Chickens with the predominant genotype (CC) showed a higher feed efficiency than chickens with the other two genotypes. The average FCR values for CC, CG, and GG genotypes were 2.20, 2.29, and 2.40, respectively (Fig. 2C). *TNFSF13B* gene expression and RFI values among different genotypes of the variant rs312433097 were also significantly different (Fig. 3B and C). At the rs312433097 locus, chickens with the TT genotype exhibited higher feed efficiency than those with the CT and CC genotypes, with RFI values of −2.37, −0.62, and 1.52, respectively (Fig. 3C). Furthermore, differences in the expression of RFXAP and FCR among individuals with different genotypes of the variant rs14804657 are shown in Fig. 2D and E. Similarly, the comparison of *TMLHE* gene expression and RFI among individuals with different genotypes of the variant rs316724231 is shown in Fig. 3D and E.

### Identification of liver differentially expressed genes (DEGs) associated with FCR and RFI

Spearman's rank-based correlation (SRC) coefficients were calculated to determine the relationship between hepatic gene expression and feed efficiency. The analysis identified 870 genes that were significantly correlated with FCR, comprising 617 positively and 253 negatively correlated genes (*P* < 0.05, Additional file 1: Table S8 and Fig. 2F). For RFI, 744 genes were found to be significantly correlated, including 82 positively and 662 negatively correlated genes (*P* < 0.05, Additional file 1: Table S9 and Fig. 3F). Additionally, we identified DEGs between individuals in the top and bottom 20% of FCR and RFI groups, resulting in 890 DEGs associated with FCR and 367 DEGs associated with RFI (*P* < 0.05, Additional file 1: Table S10, S11 and Fig. 2F, 3F). Among the FCR-related DEGs, 528 (59.3%) overlapped with genes identified through SRC analysis, including the *t﻿rans*-eGene *SUCLA2* (Fig. 2F). Similarly, among the RFI-related DEGs, 194 DEGs (52.9%) were consistent with the genes identified through SRC analysis (Fig. 3F). These findings provide a valuable dataset of DEGs associated with feed efficiency.

### Identification of key microbes related to feed efficiency

**Fig. 4:**
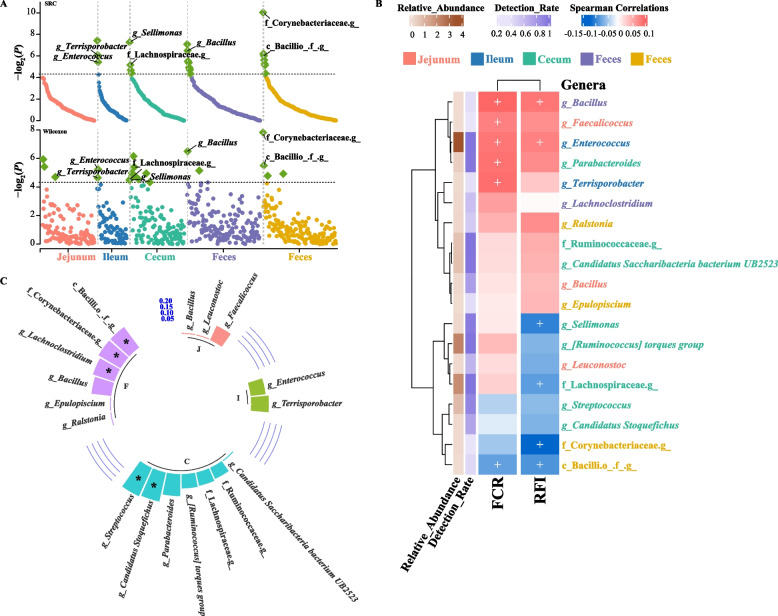
Identification of feed efficiency-related and heritable microbial taxa. **A** The upper part displays the Spearman's rank-based correlation (SRC) analysis between different microbial genera in different gut segments and FCR and RFI. The lower part shows the Wilcoxon rank-sum test of microbial abundance between chickens with the highest and lowest FCR and RFI in different gut segments. The *P* values of the correlation analysis and significance test were plotted as −log_2_(*P*). Each point represents a microbial genus, with the black dashed line indicating the significance threshold (*P* = 0.05). Green dots indicate genera that passed the significance threshold. For both correlation analysis and Wilcoxon rank-sum test, the gray dashed line represents *P*-values < 0.05. **B** The Wilcoxon rank-sum test and linear discriminant analysis Effect Size (LEfSe) analysis identified 18 differentially shared microbial genera between chickens with the highest and lowest FCR or RFI (LDA > 2). Spearman’s correlations with FCR and RFI as well as the relative abundance and detection rate for each microbial genus are provided. The plus sign indicates statistical significance *P* < 0.05. **C** Heritability estimates for the 18 differentially shared microbial genera associated with FCR and RFI in diverse segments

Based on the results of *m*^2^, we conducted SRC analysis on the abundance of microbial genera in the jejunum, ileum, cecum, and feces with respect to FCR and RFI. We performed Wilcoxon rank-sum tests and LEfSe analysis on the abundance of gut microbial genera in the top and bottom 20% of chickens ranked by FCR and RFI with the aim of identifying key differential microbes associated with feed efficiency. We identified 12 genera (3 in the ileum, 9 in the feces) associated with FCR and 15 genera (2 in the jejunum, 6 in the cecum, and 7 in the feces) associated with RFI (*P* < 0.05, Additional file 1: Table S12 and Table S13). Wilcoxon rank-sum tests and LEfSe analysis revealed 4 (2 in the ileum, 2 in the feces, Additional file 1: Table S14) and 23 genera (11 in the ileum, 12 in the feces) associated with FCR, respectively, and 20 (3 in the jejunum, 13 in the cecum, and 4 in the feces, Additional file 1: Table S15) and 36 genera (11 in the jejunum, 19 in the cecum, and 6 in the feces) associated with RFI, respectively. With the exception of three microbial genera in the cecum, the significantly different microbial genera identified through Wilcoxon rank-sum tests in each gut segment were further validated by LEfSe analysis (LDA > 2.0, *P*_adj_ < 0.05, Kruskal–Wallis test, Additional file 1: Table S16). In these associations, 3 genera related to FCR and 4 genera related to RFI were observed in both the association analysis and significance tests (Fig. 4A). Of the common genera associated with FCR, 2 (*Terrisporobacter* and *Enterococcus*) and one (*Bacillus*) were located in the ileum and feces, respectively. *Terrisporobacter* and *Enterococcus* in the ileum and *Bacillus* in the feces were significantly positively correlated with FCR (*P* < 0.05, Fig. 4B). Regarding RFI-related genera, 2 genera (*Sellimonas* and *Lachnospiraceae*) and 2 genera (*Corynebacteriaceae* and *Bacilli*) were identified in the cecum and feces, respectively. The genus *Sellimonas* and the family Lachnospiraceae in the cecum, as well as the family Corynebacteriaceae and the class Bacilli in the feces, were all significantly negatively correlated with RFI (*P* < 0.05, Fig. 4B). The detection rates of these microbial taxa were relatively high in the corresponding gut segments, all exceeding 35%. Furthermore, LEfSe analysis confirmed the differences in many other taxa related to FCR and RFI identified by Wilcoxon rank-sum tests (Additional file 1: Table S14 and S15).

### Association between host genetics and the key gut microbiota related to feed efficiency

Based on the estimation of *m*^2^, we further estimated the heritability of gut microbiota at the genus level in the jejunum, ileum, cecum and feces. The cumulative relative abundances of genera with detection rates ≥ 30% in the jejunum, ileum, cecum and feces were 91.04%, 95.01%, 97.64%, and 91.76%, respectively. Therefore, analysis of the heritability of genera with a detection rate ≥ 30% could represent the overall situation of the gut microbiota. In the jejunum, ileum, cecum and feces, 6 (4.38%), 11 (14.29%), 35 (24.82%), and 17 (9.04%) genera were found to have significant heritability, respectively (*P* < 0.05, Additional file 1: Table S17 and Additional file 4: Fig. S3). Genera with significant heritability were predominantly from the phyla Firmicutes and Proteobacteria, with the cecum dominated by the order Clostridiales. We specifically focused on those 18 genera associated with FCR and RFI through both the Wilcoxon rank-sum test and LEfSe analysis, and only 5 exhibited significant heritability (*P* < 0.05). These included the genera *Streptococcus* and *Candidatus Stoquefichus* in the cecum associated with RFI, and the family Corynebacteriaceae and the class Bacilli in the feces associated with RFI, as well as the genus *Lachnoclostridium* in the feces associated with FCR (Fig. 4C). We further conducted microbial genome-wide association studies (mGWAS) on these 18 genera and identified 3 genera that were significantly associated with genomic variants. Specifically, we found that the SNPs rs734075874 and rs318196709 on chromosome 6 were significantly associated with the abundance of the genus *Terrisporobacter* in the ileum related to FCR. Furthermore, the SNPs rs14591753, rs14591754, and 6:31040116 on chromosome 6 were significantly associated with the abundances of genus *Leuconostoc* in the jejunum, which is related to RFI. Details of the significant SNPs associated with the abundance of key microbial genera are shown in Additional File 1: Table S18.

### Identification of host genomic variants and liver eQTLs associated with gut microbiota with significant differences related to feed efficiency

We conducted SMR analysis based on summary data from liver eQTL mapping and mGWAS to estimate the causal relationship between gene expression levels and the abundance of key microbial genera. We identified 60 gene–microbe associations that were significant in the SMR test (Additional file 1: Table S19).

**Fig. 5:**
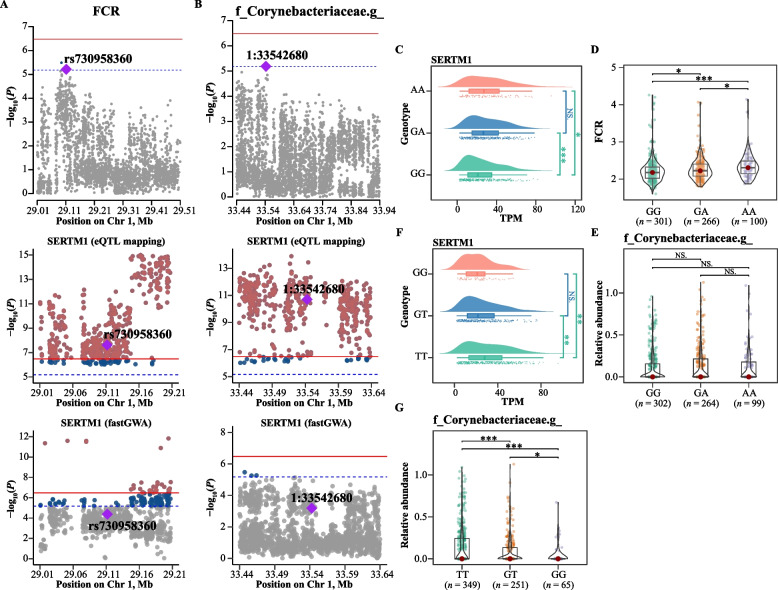
Genomic determinants of key microbial genera, feed efficiency, and their shared potential causal genes. **A** and** B** Genome-wide association analysis of the FCR and microbes. The horizontal red solid line and blue dashed line indicate genome-wide significance (*P* = 3.32 × 10^−7^) and suggestive significance (*P* = 6.63 × 10^−6^) thresholds. Gray, dark blue and reddish-brown dots indicate non-significant, suggestively significant and significant SNPs, respectively. Colocalization analysis was performed for *trans*-eQTLs of the gene *SERTM1* in chicken liver and the GWAS loci for FCR on chromosome 1, as well as the GWAS loci for the relative abundance of fecal Corynebacteriaceae on chromosome 1. The colocalized SNPs, rs730958360 and 1:33542680, which was the significant *trans*-eQTL for *SERTM1* and suggestively significant GWAS signals for both FCR and the relative abundance of fecal Corynebacteriaceae, did not reach the genome-wide significance level. **C** and** F** The expression levels of *SERTM1* in the liver corresponding to the 3 genotypes of the 2 eVariants (rs730958360 and 1:33542680) were compared. ***, **, *, and NS represent adjusted *P* values < 0.001, < 0.01, < 0.05, and > 0.05, respectively. **D** Comparison of FCR among the three genotypes of the eVariant rs730958360. Each point represents an individual chicken. The data and center red points indicate the number and median values for the corresponding genotypes, respectively. ***, **, *, and NS represent adjusted *P* values < 0.001, < 0.01, < 0.05, and > 0.05, respectively. **E** and** G** Comparison of the relative abundance of fecal Corynebacteriaceae among the three genotypes of the eVariants rs730958360 and 1:33542680. Each point represents a sample. The data and center red point respectively indicate the number and median value for the corresponding genotypes. ***, **, *, and NS represent adjusted *P* values < 0.001, < 0.01, < 0.05, and > 0.05, respectively

**Fig. 6:**
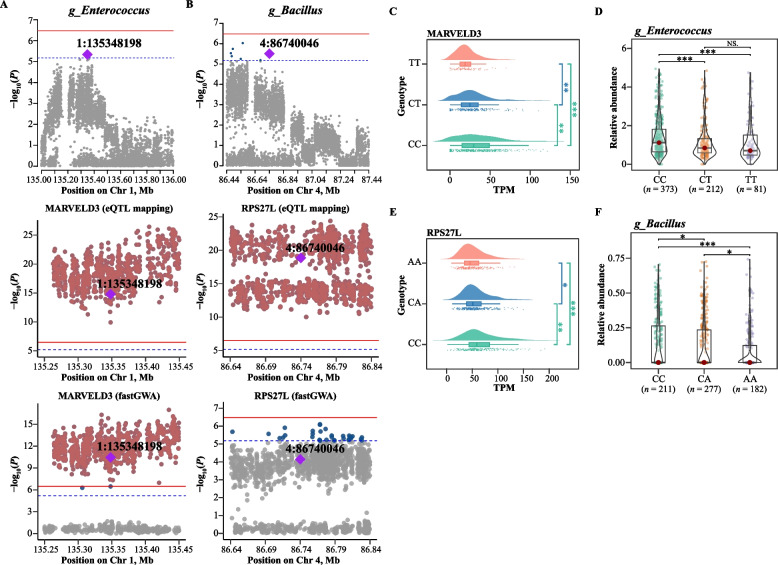
Genomic determinants of key microbial genera and their shared potential causal genes with differentially expressed genes (DEGs) related to feed efficiency. **A** and** B** Microbial genome-wide association studies. The horizontal red solid line and blue dashed line indicate genome-wide significance (*P* = 3.32 × 10^−7^) and suggestive significance (*P* = 6.63 × 10^−6^) thresholds. Gray, dark blue and reddish-brown dots indicate non-significant, suggestively significant and significant SNPs, respectively. Colocalization analysis was conducted for *trans*-eQTLs of the *MARVELD3* gene in chicken liver and GWAS loci for the relative abundance of *Enterococcus* in the ileum on chromosome 1. Similarly, colocalization analysis was performed for *trans*-eQTLs of the *RPS27L* gene in chicken liver and the GWAS loci for the relative abundance of *Bacillus* in the feces on chromosome 4. One colocalized SNP was identified, which was a significant *trans*-eQTL for *MARVELD3* and a suggestively significant GWAS signal for the relative abundance of *Enterococcus* in the ileum. Another colocalized SNP did not reach genome-wide significance, but indicated a significant *trans*-eQTL for *RPS27L* and a suggestively significant GWAS signal for the relative abundance of *Bacillus* in the feces. **C** and** E** The expression levels of *MARVELD3* and *RPS27L* genes in the liver corresponding to the 3 genotypes of these 2 eVariant (1:135348198 and 4:86740046) were compared. ***, **, *, and NS represent adjusted *P* values < 0.001, < 0.01, < 0.05, and > 0.05, respectively. **D** and** F** Comparison of the relative abundance of *Enterococcus* in the ileum among the three genotypes of eVariant 1:135348198 and the relative abundance of *Bacillus* in the feces among the three genotypes of eVariant 4:86740046. Each point represents a sample. The data and center red point respectively indicate the number and median value for the corresponding genotypes. ***, **, *, and NS represent adjusted *P* values < 0.001, < 0.01, < 0.05, and > 0.05, respectively

To further investigate the combined impact of host genetics and microbiota on feed efficiency, we intersected the gene sets obtained from SMR tests for gene‐trait associations with the gene sets obtained from SMR tests for gene‐microbe associations. Only one protein-coding gene, *SERTM1*, was concurrently located in the sets. SERTM1 exhibited significant associations with the SNPs rs730958360 (1:29111712) and 1:33542680 on GGA1 (Fig. 5A and B). At the SNP rs730958360 locus, the expression of the *SERTM1* gene in chickens with the GG genotype was significantly lower than in those with the GA and AA genotypes, and the G to A substitution led to a significant increase in FCR values (low feed efficiency) (Fig. 5C and D). However, at SNP rs730958360, the difference in the abundance of the Corynebacteriaceae family among chickens with different genotypes was not significant, and the average relative abundance of this bacterium was less than 0.2% (Fig. 5E). At SNP 1:33542680, the expression of the *SERTM1* gene in chickens with the TT genotype was significantly higher than that in those with the GT and GG genotypes, and the T to G substitution at this position resulted in a significant decrease in the abundance of the family Corynebacteriaceae (Fig. 5F and G). We also analyzed the intersection of FCR- and RFI-related DEGs with the gene sets obtained from SMR tests for gene‐microbe associations. The results revealed that a protein-coding gene, *MARVELD3,* was simultaneously identified in the DEGs related to FCR and RFI, and exhibited a significant negative correlation with both FCR and RFI. Furthermore, *MARVELD3* was significantly associated with SNP 1:135348198, and this gene was significantly correlated with the abundance of the genus *Enterococcus* in the ileum related to FCR (Fig. 6A). For SNP 1:135,348,198, the C to T substitution led to a significant decrease in the expression of the *MARVELD3* gene; at this locus, chickens with the CC genotype had a significantly higher abundance of the genus *Enterococcus* than those with CT and TT genotypes (Fig. 6C and D). Another protein-coding gene, *RPS27L*, was located in the DEGs related to FCR, and showed a significant negative correlation with FCR. *RPS27L* was significantly associated with the SNP 4:86740046 on GGA4, and this gene was significantly correlated with the abundance of the genus *Bacillus* in feces related to FCR (Fig. 6B). For SNP 4:86740046, the C to A substitution led to a significant decrease in the expression of RPS27L and the abundance of the genus *Bacillus* (Fig. 6E and F).

**Fig. 7:**
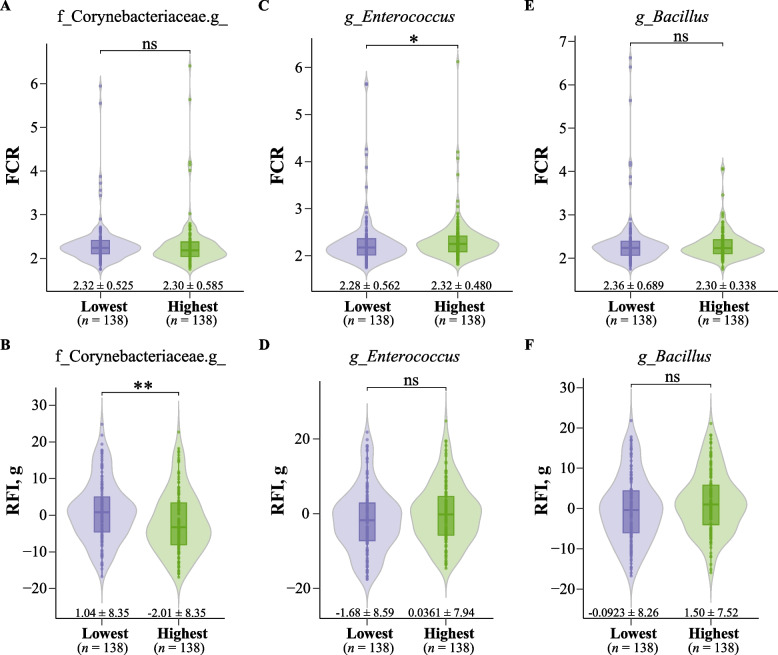
Key microbiota related to feed efficiency. Differences in FCR between the two groups with the lowest and highest abundance of **A** fecal Corynebacteriaceae, **C** ileal *Enterococcus*, and **E** fecal *Bacillus*. Differences in RFI between the two groups with the lowest and highest abundance of **B** fecal Corynebacteriaceae, **D** ileal *Enterococcus*, and **F** fecal *Bacillus*. Each point represents a sample. The data are presented as the number of individuals and the mean ± SD in the corresponding group. **, * and ns indicate adjusted *P* values < 0.01, < 0.05, and > 0.05, respectively

We focused specifically on the three key microbiotas mentioned above. The differences in FCR between the 20% of chickens with the lowest and highest abundance of Corynebacteriaceae in feces were not significant, but the RFI value of the 20% of chickens with the highest abundance of Corynebacteriaceae in feces was significantly lower than those with the lowest abundance (Fig. 7A and B). The 20% of chickens with the lowest abundance of *Enterococcus* in the ileum showed significantly lower FCR values than those with the highest abundance, whereas RFI differences were not significant (Fig. 7C and D). For both FCR and RFI, there were no significant differences between the 20% of chickens with the lowest and highest abundance of *Bacillus* in feces (Fig. 7E and F).

## Discussion

Feed efficiency is a complex trait influenced by various factors, including genetics and gut microbiota [7, 14]. Therefore, a systematic strategy is required to address the mechanisms underlying the variability in feed efficiency. Here, we employed a multi-omics approach to analyze the genomic genetic variations, gene expression, and changes in gut microbiota that influence feed efficiency in the late laying period of laying hens. This study provides unique insights into the potential regulatory mechanisms of feed efficiency in laying hens.

In poultry production, the commonly used indicators to evaluate animal feed utilization efficiency include FCR and RFI. The emphasis on selecting low FCR is to improve egg production performance without reducing feed intake, whereas the focus on selecting low RFI is to reduce individual feed consumption without decreasing egg production [63, 64]. Artificial selection can effectively enhance feed efficiency [65]. Thus, understanding the genetic background of feed efficiency is crucial for further improvement. We estimated the heritability (*h*^2^) of FCR and RFI and found that host genetics contributed to 28% and 48% of the phenotypic variation in FCR and RFI, respectively. Accumulated research in recent studies have suggested that FCR and RFI generally exhibit moderately high heritability [3, 6, 66, 67]. Taken together, these results suggest that feed efficiency is at a moderate level of genetic control. Based on summary statistics from GWAS and eQTL studies, we conducted SMR analysis and identified 4 potential causal genes for FCR and 2 for RFI. To further validate the results, fastGWA [60] was employed based on a colocalization strategy, and the genes *SUCLA2* and *TNFSF13B* were once again identified. This indicates that the results observed in the SMR analysis for FCR and RFI were robust. Additionally, SRC and ANOVA again identified *SUCLA2* to be significantly positively correlated with FCR. By analyzing the transcriptome differences between high- and low body weight lines of chickens, Jia et al. [68] identified the candidate gene *SUCLA2* located within the QTL region showing differential expression. The identified *SUCLA2* in this study is a *trans*-regulated gene, the eVariant 1:25664581 may influence feed efficiency by indirectly affecting the expression of the *SUCLA2* gene. In terms of biological mechanisms, *SUCLA2*, which encodes the ATP-specific beta isoform of succinyl-CoA synthetase (SCS), has a crucial impact on energy metabolism. SCS is an important enzyme in the tricarboxylic acid (TCA) cycle, catalyzing the conversion of succinyl-CoA into succinic acid while being coupled with the phosphorylation of ADP or GDP to produce ATP. This process is one of the main pathways for cells to generate ATP, which is crucial for maintaining cellular energy homeostasis [69, 70]. When the expression of *SUCLA2* increases, the activity of SCS also intensifies, leading to an acceleration of the TCA cycle. The accelerated TCA cycle will increase the production of metabolic energy, as more substrates (such as glucose, fatty acids, etc.) are oxidized and converted into ATP. However, this may also increase the demand for maintenance energy, as cells require more energy to sustain higher metabolic activity. From the perspective of FCR, when the expression of *SUCLA2* increases, animals may increase their feed intake to meet the increased metabolic energy demand. This is because more feed intake can ensure sufficient substrate supply for the TCA cycle, thus maintaining or enhancing the level of energy metabolism. Therefore, the significant positive correlation between *SUCLA2* and FCR may reflect the corresponding increase in feed intake in animals when energy metabolism is enhanced. TNFSF13B, also known as BAFF (B-cell Activating Factor) or TALL-1 (TNF Ligand Superfamily Member 13B), plays a pivotal role in the immune system, especially in the survival, maturation, and differentiation of B cells. Although the primary function of TNFSF13B is associated with immune responses, recent studies have shown that there is a complex interaction between the immune response and animal growth and metabolism [71, 72]. One potential mechanism is that the expression level of *TNFSF13B* may indirectly influence the RFI by affecting the activity and quantity of B cells. For instance, if the expression level of *TNFSF13B* is low, it may lead to weakened B-cell function or reduced cell counts, which may decrease the animal's immune response to pathogens or antigens. To compensate for this weakened immune response, animals may need to increase their food intake to obtain more energy and nutrients, thus enhancing the immune system and other physiological functions. Therefore, a lower expression level of *TNFSF13B* may be correlated with higher RFI. However, this mechanism is only a speculation based on current knowledge and requires further experimental validation.

In addition to host genetics, gut microbiota plays a crucial role in feed efficiency [13, 73]. Studies have suggested that variations in the gut microbiota may contribute to phenotypic differences among individuals in a population [13, 14, 74, 75]. A larger *m*^2^ value suggests that the gut microbial community provides more information regarding the studied phenotypes [13, 76–78]. Based on the *m*^2^ values, we further explored which microbial taxa in the jejunum, ileum, cecum, and feces were significantly associated with feed efficiency. Many studies have indicated that host genetic factors play a role in regulating the composition of the gut microbiome [16, 79–82]. Studies of the chicken gut microbiome have reported non-zero heritability estimates for certain microbial taxa [14, 18]. Borey et al. [83] confirmed that selective breeding can alter the composition of the gut microbiota in chickens. It is anticipated that host genetics may influence feed efficiency by either supporting or hindering microbes that make significant contributions to nutrient digestion and energy harvesting. To elucidate the genetic polymorphisms and the genes underlying the heritability of host-microbiota interactions, we conducted mGWAS on 18 significantly different microbial genera and identified genomic variations significantly associated with the abundance of the genera *Leuconostoc* in the jejunum, *Terrisporobacter* in the ileum, and *Parabacteroides* in the cecum. The genus *Leuconostoc* comprises saccharolytic bacteria that catabolize carbohydrates into lactic acid via heterolactic fermentation [84]. Miyamoto et al. [85] revealed that the exopolysaccharides (EPS) produced by *Leuconostoc* improve glucose metabolism and energy homeostasis through EPS-derived gut microbial short-chain fatty acids, and alter gut microbial composition. The genus *Terrisporobacter* has been linked to short-chain fatty acids (SCFAs) and oxidative stress in an animal study [86]. Wen et al. [14] previously reported genetic regions associated with the abundance of cecal *Parabacteroides* and found that *Parabacteroides* is one of the two more abundant genera in chickens with high RFI values (inefficient). *Parabacteroides* is involved in the regulation of host glucolipid metabolism [87, 88], Wang et al. [87] demonstrated that oral treatment with live *Parabacteroides* can reduce weight gain and improve glucose homeostasis. Therefore, these individual microbes may influence feed efficiency in various ways, including direct participation in nutrient digestion [10], fostering a mutually beneficial symbiotic relationship with the host, and maintaining intestinal health [89].

By integrating the SMR results of the microbiota with those of FCR and RFI, we found that SERTM1 is a potential causal gene associated with both FCR and the abundance of the family Corynebacteriaceae related to RFI in feces. Previous studies have indicated that *SERTM1* serves as a top marker in human islet γ-cells [90]. This demonstrates the potential significance of this gene in cell function and regulation, suggesting that *SERTM1* may exhibit similar biological functions across species. Liu et al. [91] reported a direct correlation between the *SERTM1* gene and fertility in goats. This further supports the importance of *SERTM1* in physiological processes and provides context for understanding its potential role in chickens. We hypothesized that the variant rs730958360 might influence feed efficiency by altering the expression level of *SERTM1* in the liver, whereas the variant 1:33542680 might indirectly affect feed efficiency by influencing the abundance of the family Corynebacteriaceae through the *SERTM1* gene. To date, no direct studies have reported a biological link between the *SERTM1* gene and the family Corynebacteriaceae. One conceivable rationale could be that the expression or functionality of *SERTM1* might have an impact on the physiological condition or metabolic trajectories of the host, thereby indirectly influencing the composition of the gut microbiota, specifically the abundance of Corynebacteriaceae. Our study further confirmed that a higher abundance of the Corynebacteriaceae family in feces was associated with better feed efficiency. Wen et al. [14] previously reported that a higher abundance of cecal *Corynebacterium* is related to improved feed efficiency, and this genus serves as a model genus within the family Corynebacteriaceae. Several studies have indicated a significant correlation between the abundance of the Corynebacteriaceae in the jejunum and feed efficiency [92, 93]. A previous study observed a negative correlation between the abundance of Corynebacteriaceae in the gut microbiota and average daily gain (ADG) in the obese population [94]. Additionally, we identified that *MARVELD3* is regulated by the variant 1:135348198, which affects the abundance of the genus *Enterococcus*, thereby indirectly influencing feed efficiency. These results indicate that the interaction between host genes and gut microbiota may influence feed efficiency by modulating the abundance of specific microbes. Previous studies have indicated that *MARVELD3* is a determinant of paracellular permeability in epithelial cells [95, 96]. Several studies have revealed that *MARVELD3* plays a role in epithelial-mesenchymal transition (EMT), cell proliferation, and migration [97, 98]. We inferred that MARVELD3 may lead to differences in the intestinal microenvironment, thereby affecting the colonization and growth of microorganisms. We found that lower abundance of *Enterococcus* in the ileum was associated with better feed efficiency. Previous research has suggested that *Enterococcus faecium* as an alternative to antibiotics can reduce the feed conversion rate in broiler chickens [99]. However, poultry infected with *Enterococcus* can have decreased growth rates, reduced feed efficiency, and increased mortality rates [100]. These findings suggest that selective breeding for high feed efficiency by targeting host-derived genetic variations may also influence a subset of the gut microbiota, thereby collectively contributing to the variation in feed efficiency. The antagonistic role of *Enterococcus* in the ileum on the positive effect of MARVELD3 on feed efficiency requires further investigation.

The selection strategies for FCR and RFI were different. FCR and its components are genetically dependent, making it difficult to improve without directly affecting egg production [101, 102]. In contrast, RFI is independent of growth, production, and maturation patterns [3, 103]. Our study, along with previous research [5], indicates that the genetic regulatory variants for FCR and RFI in laying hens are different. Furthermore, our study is the first to identify different gut segments related to microbial regulation and the significant microbiota for these two traits, suggesting different mechanisms of microbial regulation. To improve feed efficiency, the specific choice of indicator to use depends on the needs of the breeder.

## Conclusions

Our study indicated that both host genetics and gut microbiota contribute to feed efficiency during the late laying period in hens. Host genetics play a more important role than gut microbiota in shaping feed efficiency. The genomic variations that are potentially related to feed efficiency, including 1:25664581, rs312433097, and rs730958360, may indirectly influence feed intake and energy metabolism by affecting the expression of related genes such as *SUCLA2*, *TNFSF13B*, and *SERTM1*, which in turn may indirectly affect FCR or RFI. Similarly, the genomic variations 1:33542680 and 1:135348198 indirectly caused the changes in the microbiome, specifically related to the family Corynebacteriaceae and genus *Enterococcus*, by affecting the expression of *SERTM1* and *MARVELD3* genes. These changes may also influence the feed efficiency by affecting nutrient absorption and metabolism. Overall, our study adopted the framework of a multi-omics approach, offering a novel perspective on elucidating the molecular mechanisms underlying variations in feed efficiency in laying hens and providing crucial scientific evidence and potential starting points for developing new strategies to improve feed efficiency.

## Supplementary Information

**Additional file 1: Table S1.** Descriptive statistics for host phenotypes. **Table S2**. Summary statistics of whole-genome resequencing. **Table S3**. Summary statistics of 16S rRNA gene sequencing. **Table S4**. Detailed information on the SNPs associated with feed conversion ratio. **Table S5**. Detailed information on the SNPs associated with residual feed intake. **Table S6**. The SMR test based on the GWAS results for FCR. **Table S7**. The SMR test based on the GWAS results for RFI. **Table S8**. SRC analysis between the expression of each gene and FCR. **Table S9**. SRC analysis between the expression of each gene and RFI. **Table S10**. Differential expression genes detected between the top and bottom 20% FCR-ranked chickens. **Table S11**. Differential expression genes detected between the top and bottom 20% RFI-ranked chickens. **Table S12**. SRC analysis performed between the abundance of microbial taxa in each gut segment and FCR. **Table S13**. SRC analysis performed between the abundance of microbial taxa in each gut segment and RFI. **Table S14**. Wilcoxon rank-sum test on the relative abundance of each taxon between the top and bottom 20% FCR-ranked chickens. **Table S15**. Wilcoxon rank-sum test on the relative abundance of each taxon between the top and bottom 20% RFI-ranked chickens. **Table S16**. LEfSe analysis on the relative abundance of each taxon between the top and bottom 20% FCR/RFI-ranked chickens. **Table S17**. Heritabilityand cumulative abundance of heritable microbiota. **Table S18**. Detailed information on the significant SNPs associated with the abundance of key microbial genera. **Table S19**. The SMR test based on the mGWAS results for key microbial genera.

**Additional file 2: Fig. S1**. Correlations among all recorded phenotypes, including daily feed intake, daily egg mass, daily body weight gain, metabolic body weight, feed conversion ratioand residual feed intake. The lower panel shows scatterplots for each pair of observations. Each point represents one individual.

**Additional file 3: Fig. S2**. Number of taxa classified from phylum to species with high-quality ASVs.

**Additional file 4: Fig. S3**. Heritability estimates for microbial genera with a detection rate ≥ 30% in diverse segments.

## Data Availability

Whole-genome resequencing data are available in the NCBI Sequence Read Archive (SRA) under accession SUB13720715, and RNA-Seq data are available in the SRA under accession SUB13062074. 16S rRNA sequencing data can be accessed on SRA under the accession numbers SUB12033010 (duodenum), SUB12035295 (jejunum), SUB12035349 (ileum), SUB12035378 (cecum), and SUB12035409 (feces).

## Abbreviations

ADG: Average daily gain
ANOVA: Analysis of variance
DBWG: Daily body weight gain
DEG: Differentially expressed genes
DEM: Daily egg mass
DFI: Daily feed intake
EMT: Epithelial-mesenchymal transition
eQTL: Expression quantitative trait locus
FCR: Feed conversion ratio
GCTA: Genome-Wide Complex Trait Analysis
GRM: Genetic relationship matrix
GWAS: Genome-wide analysis study
LEfSe: Linear discriminant analysis Effect Size
LMM: Linear mixed model
MARVELD3: MARVEL domain containing 3
MBW: Metabolic body weight
mGWAS: Microbial genome-wide association studies
RFI: Residual feed intake
RFXAP: Regulatory factor X associated protein
RPS27L: Ribosomal protein S27 like
SERTM1: Serine rich and transmembrane domain containing 1
SMR: Summary data-based Mendelian randomization
SRC: Spearman's rank-based correlation
SUCLA2: Succinate-CoA ligase ADP-forming beta subunit
TMLHE: Trimethyllysine hydroxylase, epsilon
TNFSF13B: Tumor necrosis factor superfamily member 13b

## References

[1] Zhang W, Aggrey S. Genetic variation in feed utilization efficiency of meat-type chickens. Worlds Poult Sci J. 2003;59(3):328–39. 10.1079/WPS20030020. 10.1079/WPS20030020

[2] Sharma V, Kundu S, Datt C, Prusty S, Kumar M, Sontakke U. Buffalo heifers selected for lower residual feed intake have lower feed intake, better dietary nitrogen utilisation and reduced enteric methane production. J Anim Physiol Anim Nutr (Berl). 2018;102(2):e607–14. 10.1111/jpn.12802. 29027698 10.1111/jpn.12802

[3] Yuan J, Dou T, Ma M, Yi G, Chen S, Qu L, et al. Genetic parameters of feed efficiency traits in laying period of chickens. Poult Sci. 2015;94(7):1470–5. 10.3382/ps/pev122. 26009751 10.3382/ps/pev122 PMC4991064

[4] Koch RM, Swiger LA, Chambers D, Gregory KE. Efficiency of feed use in beef cattle. J Anim Sci. 1963;22(2):486–94. 10.2527/jas1963.222486x. 10.2527/jas1963.222486x

[5] Yuan J, Wang K, Yi G, Ma M, Dou T, Sun C, et al. Genome-wide association studies for feed intake and efficiency in two laying periods of chickens. Genet Sel Evol. 2015;47:82. 10.1186/s12711-015-0161-1. 10.1186/s12711-015-0161-1 PMC4608132 26475174

[6] Zhou Q, Lan F, Gu S, Li G, Wu G, Yan Y, et al. Genetic and microbiome analysis of feed efficiency in laying hens. Poult Sci. 2023;102(4):102393. 10.1016/j.psj.2022.102393. 36805401 10.1016/j.psj.2022.102393 PMC9958098

[7] Sell-Kubiak E, Wimmers K, Reyer H, Szwaczkowski T. Genetic aspects of feed efficiency and reduction of environmental footprint in broilers: a review. J Appl Genet. 2017;58(4):487–98. 10.1007/s13353-017-0392-7. 28342159 10.1007/s13353-017-0392-7 PMC5655602

[8] Bordas A, Merat P. Correlated responses in a selection experiment on residual feed-intake of adult rhode-island red cocks and hens. Ann Agric Fenn. 1984;23(4):233–7. 10.1111/1467-8675.00280. 10.1111/1467-8675.00280

[9] Tixier-Boichard M, Boichard D, Groeneveld E, Bordas A. Restricted maximum likelihood estimates of genetic parameters of adult male and female Rhode Island red chickens divergently selected for residual feed consumption. Poult Sci. 1995;74(8):1245–52. 10.3382/ps.0741245. 7479501 10.3382/ps.0741245

[10] Pan D, Yu Z. Intestinal microbiome of poultry and its interaction with host and diet. Gut Microbes. 2014;5(1):108–19. 10.4161/gmic.26945. 24256702 10.4161/gmic.26945 PMC4049927

[11] Tremaroli V, Bäckhed F. Functional interactions between the gut microbiota and host metabolism. Nature. 2012;489(7415):242–9. 10.1038/nature11552. 22972297 10.1038/nature11552

[12] Koh A, De Vadder F, Kovatcheva-Datchary P, Bäckhed F. From dietary fiber to host physiology: short-chain fatty acids as key bacterial metabolites. Cell. 2016;165(6):1332–45. 10.1016/j.cell.2016.05.041. 27259147 10.1016/j.cell.2016.05.041

[13] Yan W, Sun C, Yuan J, Yang N. Gut metagenomic analysis reveals prominent roles of Lactobacillus and cecal microbiota in chicken feed efficiency. Sci Rep. 2017;7:45308. 10.1038/srep45308. 10.1038/srep45308 PMC7365323 28349946

[14] Wen C, Yan W, Mai C, Duan Z, Zheng J, Sun C, et al. Joint contributions of the gut microbiota and host genetics to feed efficiency in chickens. Microbiome. 2021;9:126. 10.1186/s40168-021-01040-x. 34074340 10.1186/s40168-021-01040-x PMC8171024

[15] Siegerstetter S-C, Schmitz-Esser S, Magowan E, Wetzels SU, Zebeli Q, Lawlor PG, et al. Intestinal microbiota profiles associated with low and high residual feed intake in chickens across two geographical locations. PLoS ONE. 2017;12(11):e0187766. 10.1371/journal.pone.0187766. 29141016 10.1371/journal.pone.0187766 PMC5687768

[16] Goodrich JK, Waters JL, Poole AC, Sutter JL, Koren O, Blekhman R, et al. Human genetics shape the gut microbiome. Cell. 2014;159(4):789–99. 10.1016/j.cell.2014.09.053. 25417156 10.1016/j.cell.2014.09.053 PMC4255478

[17] Xie H, Guo R, Zhong H, Feng Q, Lan Z, Qin B, et al. Shotgun metagenomics of 250 adult twins reveals genetic and environmental impacts on the gut microbiome. Cell Syst. 2016;3(6):572-84. e3. 10.1016/j.cels.2016.10.004. 27818083 10.1016/j.cels.2016.10.004 PMC6309625

[18] Wen C, Yan W, Sun C, Ji C, Zhou Q, Zhang D, et al. The gut microbiota is largely independent of host genetics in regulating fat deposition in chickens. ISME J. 2019;13(6):1422–36. 10.1038/s41396-019-0367-2. 30728470 10.1038/s41396-019-0367-2 PMC6775986

[19] Rothschild D, Weissbrod O, Barkan E, Kurilshikov A, Korem T, Zeevi D, et al. Environment dominates over host genetics in shaping human gut microbiota. Nature. 2018;555(7695):210–5. 10.1038/nature25973. 29489753 10.1038/nature25973

[20] Li Q, Wang Y, Hu X, Zhang Y, Li H, Zhang Q, et al. Transcriptional states and chromatin accessibility during bovine myoblasts proliferation and myogenic differentiation. Cell Prolif. 2022;55(5):e13219. 10.1371/journal.pone.0187766. 35362202 10.1111/cpr.13219 PMC9136495

[21] Li J, Zhang D, Yin L, Li Z, Yu C, Du H, et al. Integration analysis of metabolome and transcriptome profiles revealed the age-dependent dynamic change in chicken meat. Food Res Int. 2022;156:111171. 10.1016/j.foodres.2022.111171. 35651035 10.1016/j.foodres.2022.111171

[22] Li J, Xiang Y, Zhang L, Qi X, Zheng Z, Zhou P, et al. Enhancer-promoter interaction maps provide insights into skeletal muscle-related traits in pig genome. BMC Biol. 2022;20:136. 10.1186/s12915-022-01322-2. 10.1186/s12915-022-01322-2 PMC9185926 35681201

[23] Zhang H-W, Lv C, Zhang L-J, Guo X, Shen Y-W, Nagle DG, et al. Application of omics-and multi-omics-based techniques for natural product target discovery. Biomed Pharmacother. 2021;141:111833. 10.1016/j.biopha.2021.111833. 34175822 10.1016/j.biopha.2021.111833

[24] Mohammadi-Shemirani P, Sood T, Paré G. From ‘Omics to multi-omics technologies: The discovery of novel causal mediators. Curr Atheroscler Rep. 2023;25(2):55–65. 10.1007/s11883-022-01078-8. 36595202 10.1007/s11883-022-01078-8 PMC9807989

[25] Tan X, He Z, Fahey AG, Zhao G, Liu R, Wen J. Research progress and applications of genome-wide association study in farm animals. Anim Res One Health. 2023;1(1):56–77. 10.1002/aro2.14. 10.1002/aro2.14

[26] Xu Z, Ji C, Zhang Y, Zhang Z, Nie Q, Xu J, et al. Combination analysis of genome-wide association and transcriptome sequencing of residual feed intake in quality chickens. BMC Genomics. 2016;17:594. 10.1186/s12864-016-2861-5. 27506765 10.1186/s12864-016-2861-5 PMC4979145

[27] Smith GD, Ebrahim S. ‘Mendelian randomization’: can genetic epidemiology contribute to understanding environmental determinants of disease? Int J Epidemiol. 2003;32(1):1–22. 10.1093/ije/dyg070. 12689998 10.1093/ije/dyg070

[28] Hemani G, Zheng J, Elsworth B, Wade KH, Haberland V, Baird D, et al. The MR-Base platform supports systematic causal inference across the human phenome. Elife. 2018;7:e34408. 10.7554/eLife.34408. 29846171 10.7554/eLife.34408 PMC5976434

[29] Verbanck M, Chen C-Y, Neale B, Do R. Detection of widespread horizontal pleiotropy in causal relationships inferred from Mendelian randomization between complex traits and diseases. Nat Genet. 2018;50(5):693–8. 10.1038/s41588-018-0099-7. 29686387 10.1038/s41588-018-0099-7 PMC6083837

[30] Yengo L, Sidorenko J, Kemper KE, Zheng Z, Wood AR, Weedon MN, et al. Meta-analysis of genome-wide association studies for height and body mass index in ∼700000 individuals of European ancestry. Hum Mol Genet. 2018;27(20):3641–9. 10.1093/hmg/ddy271. 30124842 10.1093/hmg/ddy271 PMC6488973

[31] Porcu E, Rüeger S, Lepik K, Santoni FA, Reymond A, Kutalik Z. Mendelian randomization integrating GWAS and eQTL data reveals genetic determinants of complex and clinical traits. Nat Commun. 2019;10:3300. 10.1038/s41467-019-10936-0. 10.1038/s41467-019-10936-0 PMC6656778 31341166

[32] Zhu Z, Zhang F, Hu H, Bakshi A, Robinson MR, Powell JE, et al. Integration of summary data from GWAS and eQTL studies predicts complex trait gene targets. Nat Genet. 2016;48(5):481–7. 10.1038/ng.3538. 27019110 10.1038/ng.3538

[33] van der Graaf A, Claringbould A, Rimbert A, consortium B, Westra H-J, Li Y, et al. A novel Mendelian randomization method identifies causal relationships between gene expression and low-density lipoprotein cholesterol levels. bioRxiv. 2019:671537. 10.1101/671537.

[34] Bain MM, Nys Y, Dunn IC. Increasing persistency in lay and stabilising egg quality in longer laying cycles. What are the challenges? Br Poult Sci. 2016;57(3):330–8. 10.1080/00071668.2016.1161727. 26982003 10.1080/00071668.2016.1161727 PMC4940894

[35] Zukiwsky N, Afrouziyeh M, Robinson F, Zuidhof M. Broiler growth and efficiency in response to relaxed maternal feed restriction. Poult Sci. 2021;100(4):100993. 10.1016/j.psj.2021.01.016. 33610891 10.1016/j.psj.2021.01.016 PMC7905470

[36] Yuan J, Chen S, Shi F, Wu G, Liu A, Yang N, et al. Genome-wide association study reveals putative role of gga-miR-15a in controlling feed conversion ratio in layer chickens. BMC Genomics. 2017;18:699. 10.1186/s12864-017-4092-9. 28877683 10.1186/s12864-017-4092-9 PMC5586008

[37] Yan W, Sun C, Wen C, Ji C, Zhang D, Yang N. Relationships between feeding behaviors and performance traits in slow-growing yellow broilers. Poult Sci. 2019;98(2):548–55. 10.3382/ps/pey424. 30239851 10.3382/ps/pey424

[38] Li H, Durbin R. Fast and accurate short read alignment with burrows-wheeler transform. Bioinformatics. 2009;25(14):1754–60. 10.1093/bioinformatics/btp324. 19451168 10.1093/bioinformatics/btp324 PMC2705234

[39] Li H, Handsaker B, Wysoker A, Fennell T, Ruan J, Homer N, et al. The Sequence Alignment/Map format and SAMtools. Bioinformatics. 2009;25(16):2078–9. 10.1093/bioinformatics/btp352. 10.1093/bioinformatics/btp352 PMC2723002 19505943

[40] McKenna A, Hanna M, Banks E, Sivachenko A, Cibulskis K, Kernytsky A, et al. The genome analysis toolkit: a MapReduce framework for analyzing next-generation DNA sequencing data. Genome Res. 2010;20(9):1297–303. 10.1101/gr.107524.110. 20644199 10.1101/gr.107524.110 PMC2928508

[41] Purcell S, Neale B, Todd-Brown K, Thomas L, Ferreira MA, Bender D, et al. PLINK: a tool set for whole-genome association and population-based linkage analyses. Am J Hum Genet. 2007;81(3):559–75. 10.1086/519795. 17701901 10.1086/519795 PMC1950838

[42] Browning SR, Browning BL. Rapid and accurate haplotype phasing and missing-data inference for whole-genome association studies by use of localized haplotype clustering. Am J Hum Genet. 2007;81(5):1084–97. 10.1086/521987. 17924348 10.1086/521987 PMC2265661

[43] Chen S, Zhou Y, Chen Y, Gu J. fastp: an ultra-fast all-in-one FASTQ preprocessor. Bioinformatics. 2018;34(17):i884–90. 10.1093/bioinformatics/bty560. 30423086 10.1093/bioinformatics/bty560 PMC6129281

[44] Kim D, Langmead B, Salzberg SL. HISAT: a fast spliced aligner with low memory requirements. Nat Methods. 2015;12(4):357–60. 10.1038/nmeth.3317. 25751142 10.1038/nmeth.3317 PMC4655817

[45] Liao Y, Smyth GK, Shi W. featureCounts: an efficient general purpose program for assigning sequence reads to genomic features. Bioinformatics. 2014;30(7):923–30. 10.1093/bioinformatics/btt656. 24227677 10.1093/bioinformatics/btt656

[46] Bolyen E, Rideout JR, Dillon MR, Bokulich NA, Abnet CC, Al-Ghalith GA, et al. Reproducible, interactive, scalable and extensible microbiome data science using QIIME 2. Nat Biotechnol. 2019;37(8):852–7. 10.1038/s41587-019-0209-9. 31341288 10.1038/s41587-019-0209-9 PMC7015180

[47] Callahan BJ, McMurdie PJ, Rosen MJ, Han AW, Johnson AJA, Holmes SP. DADA2: High-resolution sample inference from Illumina amplicon data. Nat Methods. 2016;13(7):581–3. 10.1038/nmeth.3869. 27214047 10.1038/nmeth.3869 PMC4927377

[48] Callahan BJ, McMurdie PJ, Holmes SP. Exact sequence variants should replace operational taxonomic units in marker-gene data analysis. ISME J. 2017;11(12):2639–43. 10.1038/ismej.2017.119. 28731476 10.1038/ismej.2017.119 PMC5702726

[49] Quast C, Pruesse E, Yilmaz P, Gerken J, Schweer T, Yarza P, et al. The SILVA ribosomal RNA gene database project: improved data processing and web-based tools. Nucleic Acids Res. 2012;41(D1):D590–6. 10.1093/nar/gks1219. 23193283 10.1093/nar/gks1219 PMC3531112

[50] R Core Team. R: A language and environment for statistical computing. 2013. https://www.R-project.org.

[51] Zierer J, Jackson MA, Kastenmüller G, Mangino M, Long T, Telenti A, et al. The fecal metabolome as a functional readout of the gut microbiome. Nat Genet. 2018;50(6):790–5. 10.1038/s41588-018-0135-7. 29808030 10.1038/s41588-018-0135-7 PMC6104805

[52] Yang J, Lee SH, Goddard ME, Visscher PM. GCTA: a tool for genome-wide complex trait analysis. Am J Hum Genet. 2011;88(1):76–82. 10.1016/j.ajhg.2010.11.011. 21167468 10.1016/j.ajhg.2010.11.011 PMC3014363

[53] Camarinha-Silva A, Maushammer M, Wellmann R, Vital M, Preuss S, Bennewitz J. Host genome influence on gut microbial composition and microbial prediction of complex traits in pigs. Genetics. 2017;206(3):1637–44. 10.1534/genetics.117.200782. 28468904 10.1534/genetics.117.200782 PMC5500156

[54] Difford GF, Plichta DR, Løvendahl P, Lassen J, Noel SJ, Højberg O, et al. Host genetics and the rumen microbiome jointly associate with methane emissions in dairy cows. PLoS Genet. 2018;14(10):e1007580. 10.1371/journal.pgen.1007580. 30312316 10.1371/journal.pgen.1007580 PMC6200390

[55] Difford G, Lassen J, Løvendahl P. Genes and microbes, the next step in dairy cattle breeding. In: Proceedings, EAAP–67th Annual Meeting, Belfast. Netherlands: Wageningen Academic Publishers; 2016. pp 285.

[56] Aulchenko YS, Ripke S, Isaacs A, Van Duijn CM. GenABEL: an R library for genome-wide association analysis. Bioinformatics. 2007;23(10):1294–6. 10.1093/bioinformatics/btm108. 17384015 10.1093/bioinformatics/btm108

[57] Zhou X, Stephens M. Genome-wide efficient mixed-model analysis for association studies. Nat Genet. 2012;44(7):821–4. 10.1038/ng.2310. 22706312 10.1038/ng.2310 PMC3386377

[58] Gao X, Starmer J, Martin ER. A multiple testing correction method for genetic association studies using correlated single nucleotide polymorphisms. Genet Epidemiol. 2008;32(4):361–9. 10.1002/gepi.20310. 18271029 10.1002/gepi.20310

[59] Blekhman R, Goodrich JK, Huang K, Sun Q, Bukowski R, Bell JT, et al. Host genetic variation impacts microbiome composition across human body sites. Genome Biol. 2015;16:191. 10.1186/s13059-015-0759-1. 26374288 10.1186/s13059-015-0759-1 PMC4570153

[60] Jiang L, Zheng Z, Qi T, Kemper KE, Wray NR, Visscher PM, et al. A resource-efficient tool for mixed model association analysis of large-scale data. Nat Genet. 2019;51(12):1749–55. 10.1038/s41588-019-0530-8. 31768069 10.1038/s41588-019-0530-8

[61] Taylor-Weiner A, Aguet F, Haradhvala NJ, Gosai S, Anand S, Kim J, et al. Scaling computational genomics to millions of individuals with GPUs. Genome Biol. 2019;20:228. 10.1186/s13059-019-1836-7. 10.1186/s13059-019-1836-7 PMC6823959 31675989

[62] Barbeira AN, Bonazzola R, Gamazon ER, Liang Y, Park Y, Kim-Hellmuth S, et al. Exploiting the GTEx resources to decipher the mechanisms at GWAS loci. Genome Biol. 2021;22:49. 10.1186/s13059-020-02252-4. 33499903 10.1186/s13059-020-02252-4 PMC7836161

[63] Herd RM, Arthur PF. Physiological basis for residual feed intake. J Anim Sci. 2009;87(14):E64–71. 10.2527/jas.2008-1345. 19028857 10.2527/jas.2008-1345

[64] Flock DK. Genetic-economic aspects of feed efficiency in laying hens. Worlds Poult Sci J. 1998;54(3):225–39. 10.1079/WPS19980015. 10.1079/WPS19980015

[65] Thiruvenkadan A, Panneerselvam S, Prabakaran R. Layer breeding strategies: an overview. Worlds Poult Sci J. 2010;66(3):477–502. 10.1017/S0043933910000553. 10.1017/S0043933910000553

[66] Wolc A, Arango J, Jankowski T, Settar P, Fulton JE, O’Sullivan NP, et al. Pedigree and genomic analyses of feed consumption and residual feed intake in laying hens. Poult Sci. 2013;92(9):2270–5. 10.3382/ps.2013-03085. 23960108 10.3382/ps.2013-03085

[67] Rowland K, Ashwell CM, Persia ME, Rothschild MF, Schmidt C, Lamont SJ. Genetic analysis of production, physiological, and egg quality traits in heat-challenged commercial white egg-laying hens using 600k SNP array data. Genet Sel Evol. 2019;51:31. 10.1186/s12711-019-0474-6. 10.1186/s12711-019-0474-6 PMC6593552 31238874

[68] Jia X, Lin H, Nie Q, Zhang X, Lamont SJ. A short insertion mutation disrupts genesis of miR-16 and causes increased body weight in domesticated chicken. Sci Rep. 2016;6:36433. 10.1038/srep36433. 10.1038/srep36433 PMC5093740 27808177

[69] Lambeth DO, Tews KN, Adkins S, Frohlich D, Milavetz BI. Expression of two succinyl-CoA synthetases with different nucleotide specificities in mammalian tissues. J Biol Chem. 2004;279(35):36621–4. 10.1074/jbc.M406884200. 15234968 10.1074/jbc.M406884200

[70] Lancaster MS, Kim B, Doud EH, Tate MD, Sharify AD, Gao HY, et al. Loss of succinyl-CoA synthetase in mouse forebrain results in hypersuccinylation with perturbed neuronal transcription and metabolism. Cell Rep. 2023;42(10):113241. 10.1016/j.celrep.2023.113241. 10.1016/j.celrep.2023.113241 PMC10683835 37819759

[71] Jung J, Zeng H, Horng T. Metabolism as a guiding force for immunity. Nat Cell Biol. 2019;21(1):85–93. 10.1038/s41556-018-0217-x. 30602764 10.1038/s41556-018-0217-x

[72] Nikkanen J, Leong YA, Krause WC, Dermadi D, Maschek JA, Van Ry T, et al. An evolutionary trade-off between host immunity and metabolism drives fatty liver in male mice. Science. 2022;378(6617):290–5. 10.1126/science.abn9886. 36264814 10.1126/science.abn9886 PMC9870047

[73] Stanley D, Hughes RJ, Geier MS, Moore RJ. Bacteria within the gastrointestinal tract microbiota correlated with improved growth and feed conversion: challenges presented for the identification of performance enhancing probiotic bacteria. Front Microbiol. 2016;7:187. 10.3389/fmicb.2016.00187. 26925052 10.3389/fmicb.2016.00187 PMC4760072

[74] Shah TM, Patel JG, Gohil TP, Blake DP, Joshi CG. Host transcriptome and microbiome interaction modulates physiology of full-sibs broilers with divergent feed conversion ratio. NPJ Biofilms Microbiomes. 2019;5(1):24. 10.1038/s41522-019-0096-3. 31552140 10.1038/s41522-019-0096-3 PMC6754422

[75] Li F, Hitch TC, Chen Y, Creevey CJ, Guan LL. Comparative metagenomic and metatranscriptomic analyses reveal the breed effect on the rumen microbiome and its associations with feed efficiency in beef cattle. Microbiome. 2019;7:6. 10.1186/s40168-019-0618-5. 10.1186/s40168-019-0618-5 PMC6332916 30642389

[76] Svihus B. Function of the digestive system. J Appl Poult Res. 2014;23(2):306–14. 10.3382/japr.2014-00937. 10.3382/japr.2014-00937

[77] Metzler-Zebeli B, Magowan E, Hollmann M, Ball M, Molnár A, Witter K, et al. Differences in intestinal size, structure, and function contributing to feed efficiency in broiler chickens reared at geographically distant locations. Poult Sci. 2018;97(2):578–91. 10.3382/ps/pex332. 29253222 10.3382/ps/pex332

[78] Díaz-Sánchez S, Perrotta AR, Rockafellow I, Alm EJ, Okimoto R, Hawken R, et al. Using fecal microbiota as biomarkers for predictions of performance in the selective breeding process of pedigree broiler breeders. PLoS ONE. 2019;14(5):e0216080. 10.1371/journal.pone.0216080. 31063485 10.1371/journal.pone.0216080 PMC6504170

[79] Li F, Li C, Chen Y, Liu J, Zhang C, Irving B, et al. Host genetics influence the rumen microbiota and heritable rumen microbial features associate with feed efficiency in cattle. Microbiome. 2019;7:92. 10.1186/s40168-019-0699-1. 31196178 10.1186/s40168-019-0699-1 PMC6567441

[80] Lu D, Tiezzi F, Schillebeeckx C, McNulty NP, Schwab C, Shull C, et al. Host contributes to longitudinal diversity of fecal microbiota in swine selected for lean growth. Microbiome. 2018;6:4. 10.1186/s40168-017-0384-1. 10.1186/s40168-017-0384-1 PMC5755158 29301569

[81] Kers JG, Velkers FC, Fischer EA, Hermes GD, Stegeman JA, Smidt H. Host and environmental factors affecting the intestinal microbiota in chickens. Front Microbiol. 2018;9:235. 29503637 10.3389/fmicb.2018.00235 PMC5820305

[82] Yang H, Wu J, Huang X, Zhou Y, Zhang Y, Liu M, et al. ABO genotype alters the gut microbiota by regulating GalNAc levels in pigs. Nature. 2022;606(7913):358–67. 10.1038/s41586-022-04769-z. 35477154 10.1038/s41586-022-04769-z PMC9157047

[83] Borey M, Estellé J, Caidi A, Bruneau N, Coville JL, Hennequet-Antier C, et al. Broilers divergently selected for digestibility differ for their digestive microbial ecosystems. PLoS ONE. 2020;15(5):e0232418. 10.1371/journal.pone.0232418. 32421690 10.1371/journal.pone.0232418 PMC7233591

[84] Raimondi S, Candeliere F, Amaretti A, Costa S, Vertuani S, Spampinato G, et al. Phylogenomic analysis of the genus Leuconostoc. Front Microbiol. 2022;13:897656. 10.3389/fmicb.2022.897656. 35958134 10.3389/fmicb.2022.897656 PMC9358442

[85] Miyamoto J, Shimizu H, Hisa K, Matsuzaki C, Inuki S, Ando Y, et al. Host metabolic benefits of prebiotic exopolysaccharides produced by Leuconostoc mesenteroides. Gut Microbes. 2023;15(1):2161271. 10.1080/19490976.2022.2161271. 36604628 10.1080/19490976.2022.2161271 PMC9828693

[86] Li H, Shang Z, Liu X, Qiao Y, Wang K, Qiao J. Clostridium butyricum alleviates enterotoxigenic *Escherichia coli* K88-induced oxidative damage through regulating the p62-Keap1-Nrf2 signaling pathway and remodeling the cecal microbial community. Front Immunol. 2021;12:771826. 10.3389/fimmu.2021.771826. 10.3389/fimmu.2021.771826 PMC8660075 34899723

[87] Wang K, Liao M, Zhou N, Bao L, Ma K, Zheng Z, et al. Parabacteroides distasonis alleviates obesity and metabolic dysfunctions via production of succinate and secondary bile acids. Cell Rep. 2019;26(1):222–35. 10.1016/j.celrep.2018.12.028. 30605678 10.1016/j.celrep.2018.12.028

[88] Wu T-R, Lin C-S, Chang C-J, Lin T-L, Martel J, Ko Y-F, et al. Gut commensal parabacteroides goldsteinii plays a predominant role in the anti-obesity effects of polysaccharides isolated from Hirsutella sinensis. Gut. 2019;68(2):248–62. 10.1136/gutjnl-2017-315458. 30007918 10.1136/gutjnl-2017-315458

[89] Hooper LV. OPINION Do symbiotic bacteria subvert host immunity? Nat Rev Microbiol. 2009;7(5):367–74. 10.1038/nrmicro2114. 19369952 10.1038/nrmicro2114

[90] van Gurp L, Fodoulian L, Oropeza D, Furuyama K, Bru-Tari E, Vu AN, et al. Generation of human islet cell type-specific identity genesets. Nat Commun. 2022;13:2020. 10.1038/s41467-022-29588-8. 10.1038/s41467-022-29588-8 PMC9019032 35440614

[91] Liu Y, Zhou Z, He X, Tao L, Jiang Y, Lan R, et al. Integrated analyses of miRNA-mRNA expression profiles of ovaries reveal the crucial interaction networks that regulate the prolificacy of goats in the follicular phase. BMC Genomics. 2021;22:812. 10.1186/s12864-021-08156-2. 34763659 10.1186/s12864-021-08156-2 PMC8582148

[92] Freetly HC, Dickey A, Lindholm-Perry AK, Thallman RM, Keele JW, Foote AP, et al. Digestive tract microbiota of beef cattle that differed in feed efficiency. J Anim Sci. 2020;98(2):skaa008. 10.1093/jas/skaa008. 31930312 10.1093/jas/skaa008 PMC7297442

[93] Myer P, Wells J, Smith T, Kuehn L, Freetly H. Microbial community profiles of the jejunum from steers differing in feed efficiency. J Anim Sci. 2016;94(1):327–38. 10.2527/jas.2015-9839. 26812338 10.2527/jas.2015-9839

[94] Palomo-Buitrago ME, Sabater-Masdeu M, Moreno-Navarrete JM, Caballano-Infantes E, Arnoriaga-Rodríguez M, Coll C, et al. Glutamate interactions with obesity, insulin resistance, cognition and gut microbiota composition. Acta Diabetol. 2019;56:569–79. 10.1007/s00592-019-01313-w. 30888539 10.1007/s00592-019-01313-w

[95] Steed E, Rodrigues NT, Balda MS, Matter K. Identification of MarvelD3 as a tight junction-associated transmembrane protein of the occludin family. BMC Cell Biol. 2009;10:95. 10.1186/1471-2121-10-95. 20028514 10.1186/1471-2121-10-95 PMC2805614

[96] Liang GH, Weber CR. Molecular aspects of tight junction barrier function. Curr Opin Pharmacol. 2014;19:84–9. 10.1016/j.coph.2014.07.017. 25128899 10.1016/j.coph.2014.07.017 PMC4330960

[97] Kojima T, Takasawa A, Kyuno D, Ito T, Yamaguchi H, Hirata K, et al. Downregulation of tight junction-associated MARVEL protein marvelD3 during epithelial–mesenchymal transition in human pancreatic cancer cells. Exp Cell Res. 2011;317(16):2288–98. 10.1016/j.yexcr.2011.06.020. 21763689 10.1016/j.yexcr.2011.06.020

[98] Steed E, Elbediwy A, Vacca B, Dupasquier S, Hemkemeyer SA, Suddason T, et al. velD3 couples tight junctions to the MEKK1–JNK pathway to regulate cell behavior and survival. J Cell Biol. 2014;204(5):821–38. 10.1083/jcb.201304115. 24567356 10.1083/jcb.201304115 PMC3941049

[99] He Y, Liu X, Dong Y, Lei J, Ito K, Zhang B. *Enterococcus faecium* PNC01 isolated from the intestinal mucosa of chicken as an alternative for antibiotics to reduce feed conversion rate in broiler chickens. Microb Cell Fact. 2021;20:122. 10.1186/s12934-021-01609-z. 10.1186/s12934-021-01609-z PMC8240220 34182992

[100] Jung A, Rautenschlein S. Comprehensive report of an *Enterococcus cecorum* infection in a broiler flock in Northern Germany. BMC Vet Res. 2014;10:311. 10.1186/s12917-014-0311-7. 10.1186/s12917-014-0311-7 PMC4297365 25539747

[101] Aggrey SE, Karnuah AB, Sebastian B, Anthony NB. Genetic properties of feed efficiency parameters in meat-type chickens. Genet Sel Evol. 2010;42:25. 10.1186/1297-9686-42-25. 10.1186/1297-9686-42-25 PMC2901204 20584334

[102] Prakash A, Saxena VK, Singh MK. Genetic analysis of residual feed intake, feed conversion ratio and related growth parameters in broiler chicken: a review. Worlds Poult Sci J. 2020;76(2):304–17. 10.1080/00439339.2020.1735978. 10.1080/00439339.2020.1735978

[103] Shirali M, Varley PF, Jensen J. Bayesian estimation of direct and correlated responses to selection on linear or ratio expressions of feed efficiency in pigs. Genet Sel Evol. 2018;50:50. 10.1186/s12711-018-0403-0. 29925306 10.1186/s12711-018-0403-0 PMC6011546

