# Supplementary figures and images for: Host genetics and gut microbiota synergistically regulate feed utilization in egg-type chickens
Source: J Anim Sci Biotechnol. 2024 Sep 9;15:123. doi: 10.1186/s40104-024-01076-7 (PMC11382517; doi:10.1186/s40104-024-01076-7)

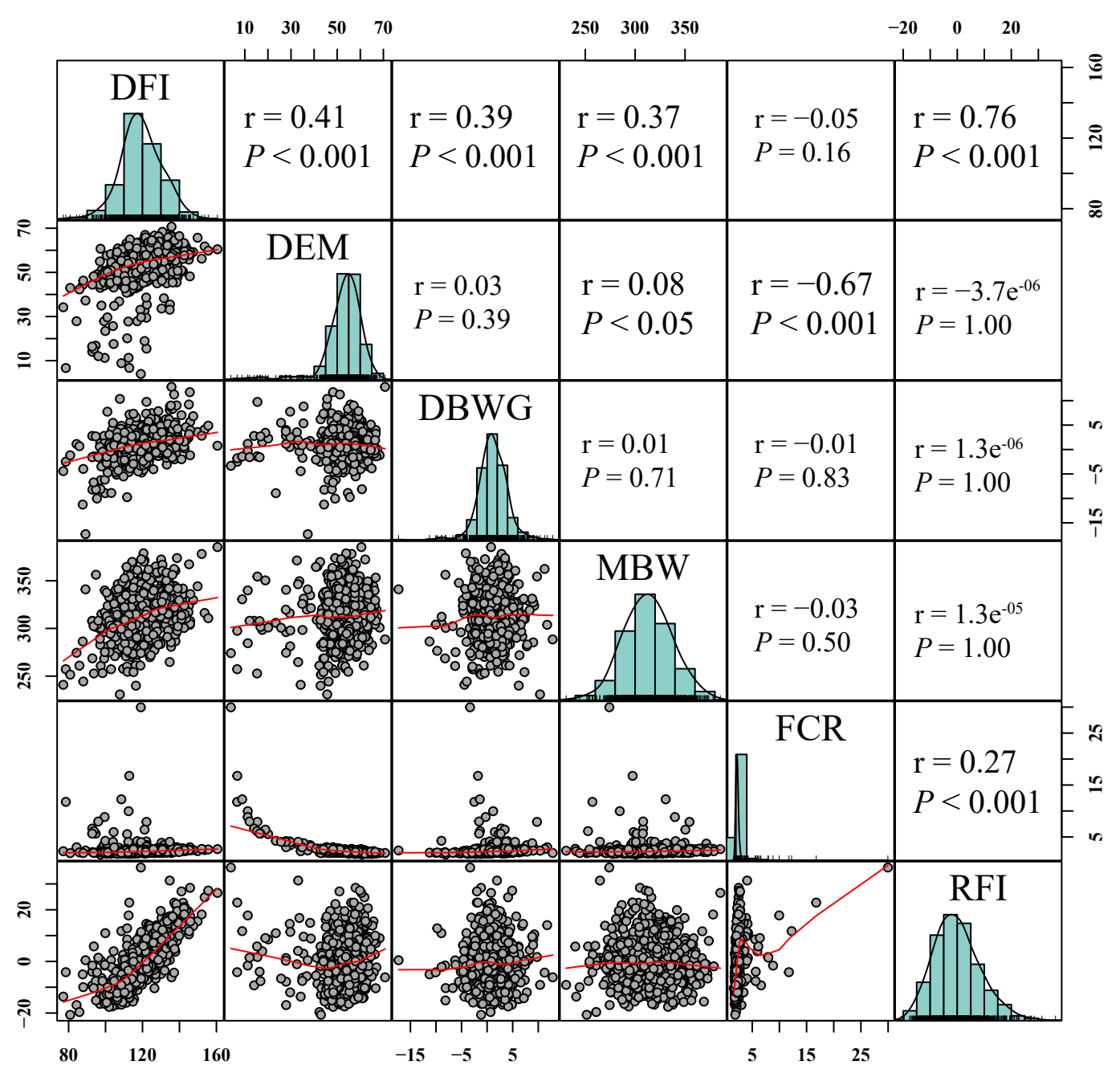

Supplement: Supplementary file 2 — Additional file 2: Fig. S1. Correlations among all recorded phenotypes, including daily feed intake, daily egg mass, daily body weight gain, metabolic body weight, feed conversion ratioand residual feed intake. The lower panel shows scatterplots for each pair of observations. Each point represents one individual. [file 40104_2024_1076_MOESM2_ESM.pdf]

Microbial genera in diverse segments

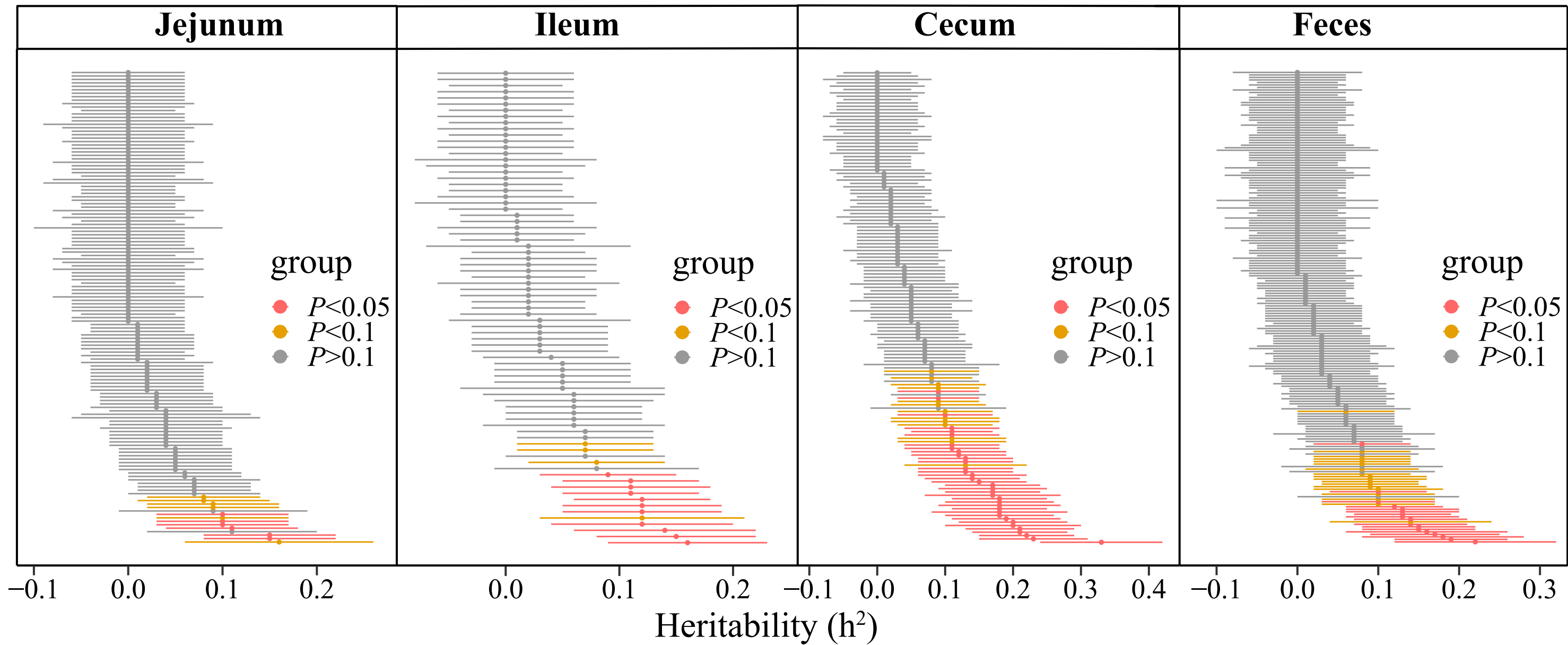

Supplement: Supplementary file 4 — Additional file 4: Fig. S3. Heritability estimates for microbial genera with a detection rate ≥ 30% in diverse segments. [file 40104_2024_1076_MOESM4_ESM.pdf]
