# Supplementary material for: Host genetics and gut microbiota synergistically regulate feed utilization in egg-type chickens
Source: J Anim Sci Biotechnol. 2024 Sep 9;15:123. doi: 10.1186/s40104-024-01076-7 (PMC11382517; doi:10.1186/s40104-024-01076-7)
Supplement: Supplementary file 3 — Additional file 3: Fig. S2. Number of taxa classified from phylum to species with high-quality ASVs. [file 40104_2024_1076_MOESM3_ESM.pdf]

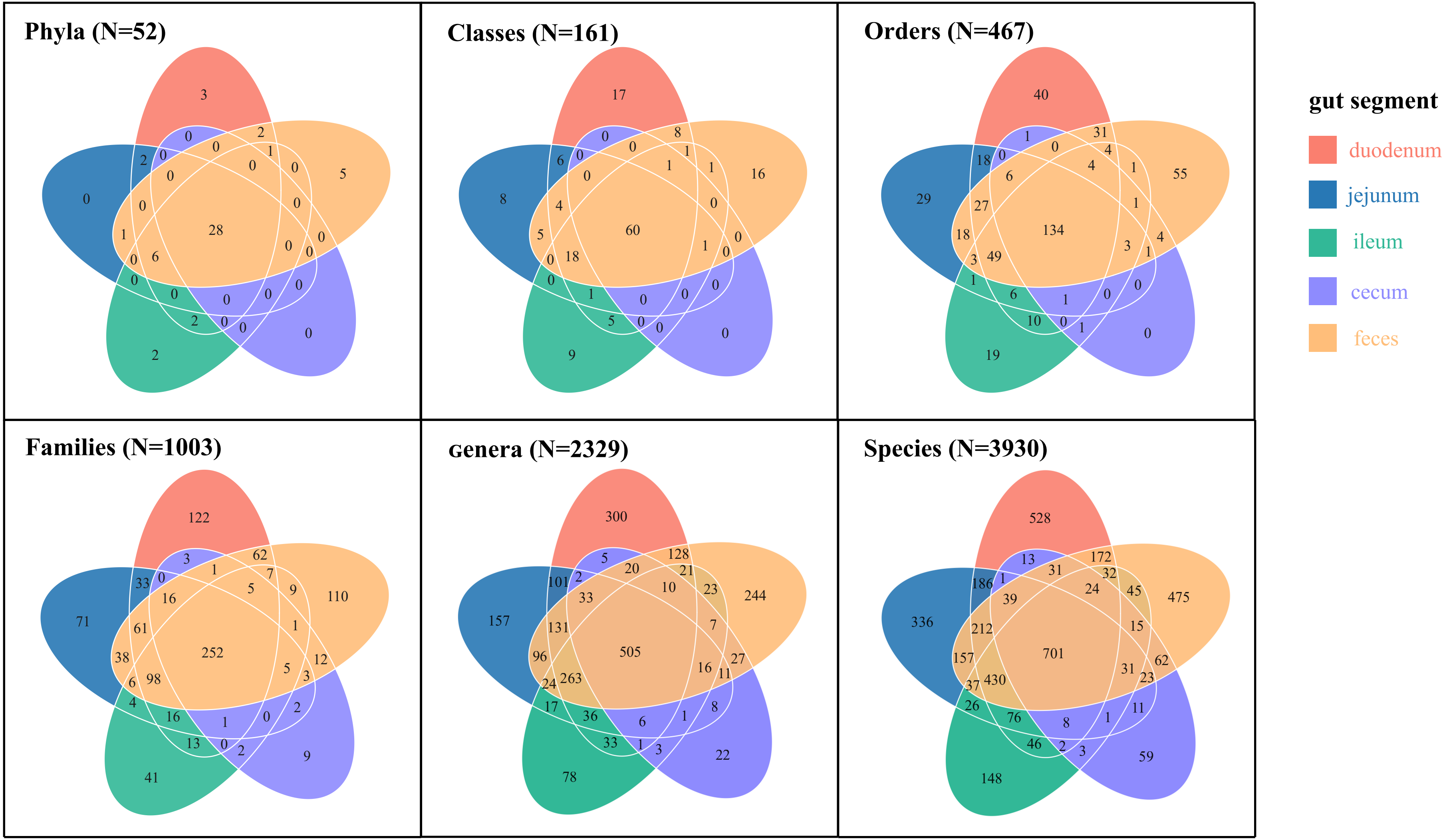
Phyla (N=52)
Classes (N=161)
Orders (N=467)
Families (N=1003)
Genera (N=2329)
Species (N=3930)
gut segment
duodenum
jejunum
ileum
cecum
feces
